# Finding a roadmap to achieve large neuromorphic hardware systems

**DOI:** 10.3389/fnins.2013.00118

**Published:** 2013-09-10

**Authors:** Jennifer Hasler, Bo Marr

**Affiliations:** School of Electrical and Computer Engineering, Georgia Institute of TechnologyAtlanta, GA, USA

**Keywords:** FPAA, Simulink, reconfigurable analog, neuromorphic engineering

## Abstract

Neuromorphic systems are gaining increasing importance in an era where CMOS digital computing techniques are reaching physical limits. These silicon systems mimic extremely energy efficient neural computing structures, potentially both for solving engineering applications as well as understanding neural computation. Toward this end, the authors provide a glimpse at what the technology evolution roadmap looks like for these systems so that Neuromorphic engineers may gain the same benefit of anticipation and foresight that IC designers gained from Moore's law many years ago. Scaling of energy efficiency, performance, and size will be discussed as well as how the implementation and application space of Neuromorphic systems are expected to evolve over time.

A primary goal since the early days of neuromorphic hardware research has been to build large-scale systems, although only recently have enough technological breakthroughs been made to allow such visions to be possible. What many people outside looking into the neuromorphic community want to see, as well as some even within the community, is the long-term technical potential and capability of these approaches. Neuromorphic engineering builds artificial systems utilizing basic nervous system operations implemented through bridging fundamental physics of the two mediums, enabling *both* superior synthetic application performance *as well as* physics and computation biological nervous systems knowledge. The particular technology choice is flexible, although most research progress is built upon analog and digital IC technologies.

Given the community is making its first serious approaches toward large-scale neuromorphic hardware [e.g., FACETs (Schemmel et al., 2008a), DARPA SyNAPSE, Caviar (Serrano-Gotarredona et al., 2009)], a neuromorphic hardware roadmap could be seen as a way through the foreseen upcoming bottlenecks (Marr et al., 2012) in computing performance, further enabling research and applications in these areas. To ignore a long-term neuromorphic approach, such as depending solely on digital supercomputing techniques, is to ignore major contemporary issues such as system power, area, and cost and misses both application opportunities as well as misses utilizing the similarities between silicon and neurobiology to drive further modeling advances.

Figure 1 shows the estimated peak computational energy efficiency for digital systems, analog signal processing, and potential neuromorphic hardware-based algorithms; we discuss the details throughout this paper. This comparison requires keeping communication local and low event rate, two properties seen in cortical structures. Computational power efficiency for biological systems is 8–9 orders of magnitude higher (better) than the power efficiency wall for digital computation; one topic this paper will explore is that analog techniques at a 10 nm node can potentially reach this same level of biological computational efficiency. Figure 1 show huge potential for neuromorphic systems, showing the community has a lot of room left for improvement, as well as potential directions on how to achieve these approaches with technology already being developed; new technologies only improve the probability of this potential being reached.

**Figure 1:**
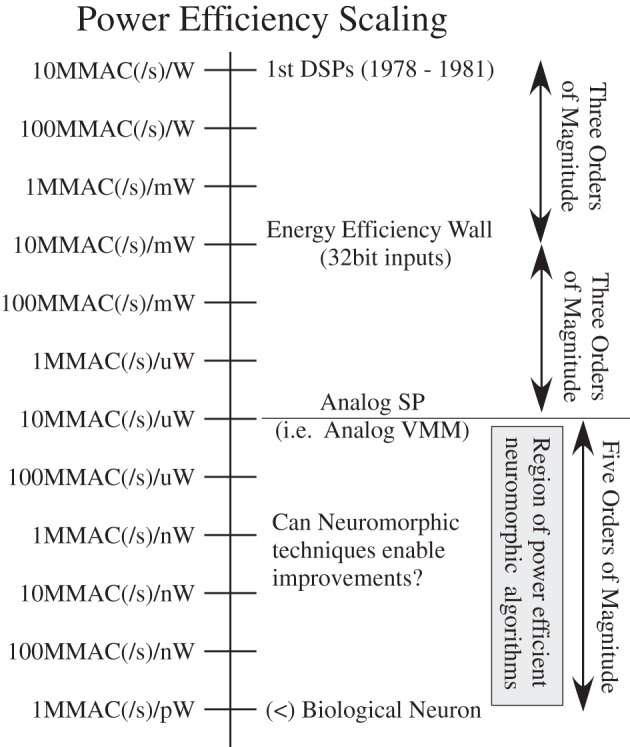
**A spectrum showing the computational efficiency of various technologies, including digital technologies, analog Signal Processing (SP), as well as best estimate of biological neuron computation.** Three orders of magnitude has produced amazing improvements in digital technology from speak-and-spell devices (Frantz and Wiggins, 1982) to current day smart phones. Three orders of magnitude in analog SP approaches has the promise of similar advancements as it becomes a stable capability. Biological neurons show a potential of five more orders of magnitude of improvement, opening further opportunity for efficient computational devices. Further, this observation defines one definition for efficient neuromorphic systems as those physically implemented algorithms that improve power efficiency beyond the analog SP metrics.

One focus is looking at what neural systems to date have a chance to scale to larger sizes, which is one metric of the particular implementation's merit going forward. In addition, considerable time is spent discussing systems that can scale and how they will be able to scale to larger systems, both in IC process improvements, circuit approaches, as well as system level constraints. One conclusions drawn is that with current research capabilities, with additional research to transition these approaches to more typical IC and system building, that reaching a system at the scale of the human brain is quite possible. Within our current grasp are circuits and technologies that can reach these large levels; when researchers are building small prototypes, these issues must be considered to enable scaling to these larger levels.

In the following sections, we will, in turn, discuss these aspects by focusing on key issues that effect this performance. section 1 will discuss a framework for discussing large-scale neuromorphic systems. section 2 discusses computational complexity and the necessary programmability and configurability, utilizing the right set of dense features to make an efficient implementation. section 3 considers the power constraints in computation and communication required to operate such systems, as well as discuss power constrained cortical structure design. section 4 continues with other key aspects to the neuromorphic roadmap, including SNR and Tools for design. We finally discuss in section 5 some initial thoughts on learning of parameters (i.e., synapses), although a complete discussion would be fairly long and complicated by the early state of research in these fields. Eventually, any useful neuromorphic system will have to rely on learning to realistically set the entire state of the network.

## Large-scale neuromorphic systems

Although the eventual goal would be the complexity of human brain, it remains beneficial to consider intermediate steps as well, such as a limited region of cortex, or potentially smaller nervous systems like a mouse. Estimates of the number of neurons in the human brain are between 10^11^ and 10^12^ (Williams and Herrup, 1988; Mead, 1990; Azevedo et al., 2009), although most recent data leans toward 10^11^ (Azevedo et al., 2009). Estimates on the number of neurons in a mouse is roughly 10^8^ neurons (Williams, 2000). Size of the cortex structure would be somewhat proportional to the sensor size of the incoming signals (Allman, 2000); size of the cortex tends to be correlated to the body size in mammals (Allman, 2000). Further, building a cortex or cortex in a handheld device imposes additional significant constraints in area and power consumed.

A lot of previous work has focused on front-end sensory and motor systems, including retina models (e.g., Mead, 1989; Boahen and Andreou, 1992; Delbruck and Mead, 1994; Delbruck, 1994; Marwick and Andreou, 2006; Lichtsteiner et al., 2008) cochlea models (e.g., Mead, 1989; van Schaik et al., 1996; Sarpeshkar et al., 1998, 2005a,b; Ravindran et al., 2005; Hamilton et al., 2008; Odame and Hasler, 2008; Rumberg et al., 2008; van Schaik et al., 2010; Rumberg and Graham, 2012) as well as others (e.g., LeMoncheck, 1992). Although these input representations are important for neural computation, and some have done some interesting engineering work based on these front-end systems (Riesenhuber and Poggio, 2000; Fu et al., 2008; Schaik et al., 2009; Chakrabartty and Liu, 2010; Liu and Delbruck, 2010; Farabet et al., 2011; Sejnowski and Delbruck, 2012), our focus will be on the computation using these front-end structures in the highly modular cortical structure (Eliasmith and Anderson, 2003).

### Si technologies for implementation: programmability, and configurability

The VLSI revolution for digital computation allowed abstraction and thus specialization in building different aspects of systems such that each group could communicate with each other and effectively contribute to the solution (Mead and Conway, 1980). This approach enabled application engineers to use digital techniques without having to be circuit or device physics experts, and as a result, rapidly increased the pace of innovation. For commercial digital IC and system development, almost all solutions are microprocessors (μP) that have become diverse in their specializations such as in digital signal processing (DSP), graphics processing (GPU), or field programmable gate arrays (FPGA). Rarely are custom IC solutions built because of the resulting cost of the mask sets and engineering time versus the projected commercial value (i.e., revenue) of the resulting solution. This direction puts more pressure on abstraction and tools for building these systems, particularly tools that enable engineering of systems as well as scientific explorations. Neuromorphic solutions utilize digital solutions where ever appropriate and effective for the resulting metrics.

Economics dictate that custom digital design at modern process nodes is typically not feasible unless there is an extremely high utilization or expected product volume, and a similar result is expected for computational approaches that are physically (biologically) inspired. The early analog VLSI research steps required heavy custom IC design to initially develop the field. On the other hand to compete either in the current signal processing, neural modeling or application development arena, analog VLSI, particularly for neuromorphic areas, must move to similar high use approaches and allow efficient programmability, configurability, and adaptability. Rarely are custom ICs built currently without high IC reuse to offset the resulting high opportunity cost. Most current approaches, heavily use digital interfacing, computation, and memories to achieve these approaches even for analog computation approaches; other efforts include researchers using long-term analog memory devices.

Physically based computation schemes, similar to analog computing, required time to develop the infrastructure for analog signal processing, neuromorphic hardware, as well as comparison with modeling approaches. These physically based solutions are inspired by the potential improvement in power efficiency and density efficiency compared to digital solutions, as well as the belief there is similar physics in Si and biological systems; more will be discussed in the following section.

**Figure 2:**
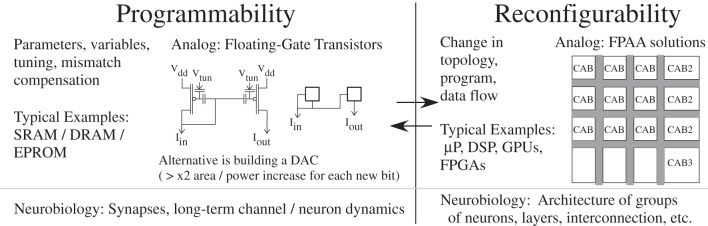
**Programmable and configurable concepts exist, recently with analog approaches as well as digital.** The line can in some cases be blurry between programmability and configurability, although for classical engineering systems the difference is fairly clear. Both concepts are critical for neurobiological systems which use programmability and configurability, sometimes in the same device. In all cases, having a long-term memory element is critical for these implementations. For analog approaches, the best solution to date has been using floating-gate based devices. Floating-gate elements are fabricated in standard CMOS, with 10 year qualified data retention for analog quantities with more than billions of read-write operations. A drop of 1–100 μV in floating-gate voltage over 10 year lifetime is typical, depending on particular process. Configurability in analog computation has seen success using Field Programmable Analog Arrays (FPAA), because analog computation is typically performed as a data-flow architecture.

Similar to how FPGAs revolutionized digital prototyping efforts, developing reconfigurable hardware that reduces the development and test cycle will fuel key innovation in neuromorphic systems. This approach requires developing configurability, which allows different computational flows, and programmability, which allows different parameter values, for physical computation systems. Figure 2 summarizes these concepts. If all values are known ahead of time, programmability is extremely useful to eliminate mismatch effects. In cases where learning is used, there is a need for parameters and precise elements. At a high level, some level of modular computing is expected given what appears to be a repeatable structure throughout cortex, thus lending itself to a configurable approach.

The most critical issue for achieving programmability and configurability in any physical computation based technology is a storage medium that enables efficient computation. The Single Transistor Learning Synapse (STLS) concept (Hasler et al., 1995) provided such an approach. The STLS are modified EEPROM devices, fabricated in a standard CMOS process, that simultaneously provide long-term storage (non-volatile), computation, and adaptation in a single device. The development of Large-Scale Field Programmable Analog Arrays (FPAA) enabled configuration to be used for physically based neuromorphic techniques (Twigg et al., 2007; Basu et al., 2010a,b; Schlottmann et al., 2010, 2012a,b, c; Wunderlich et al., 2012). These approaches allow the added advantage of those building applications not to have expertise in IC design, a separation that should prove useful for the neuromorphic community as well. General FPAA chips will be advantageous for moderate size system investigation; when structures are understood well, one would specialize some of the infrastructure, but always enable some configurability in the system. All of these aspects should enable neuromorphic/analog solutions to compete effectively with classical engineering solutions.

### Neural structure basics

One neuromorphic area focuses on building arrays of neuron elements with realistic soma dynamics at a density that enables looking at neural dynamics of 100 neurons or more (Indiveri et al., 2001; Lin et al., 2006; Renaud et al., 2007; Silver et al., 2007; Schemmel et al., 2008a; Saighi et al., 2010). Typically a tradeoff is seen between dense circuit structures and accurate modeling of biological behavior, similar to computational neuroscience but with different rules. The hope is not simply modeling neural systems, but enabling engineering applications based upon neuromorphic techniques.

**Figure 3:**
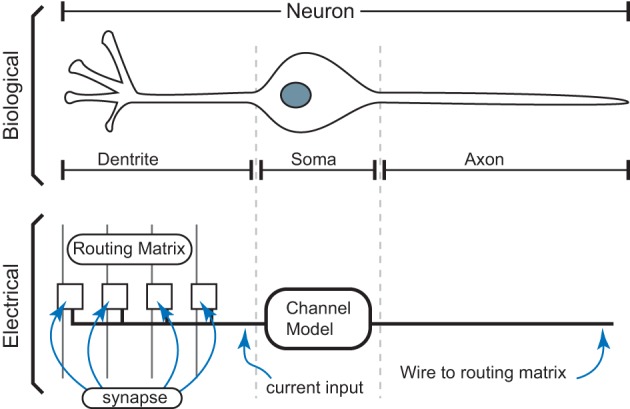
**Basic definition of neurons that uses biologically realistic transistor based models of neurobiological computation.** A biological neuron is made up of its soma, dendrite, synapse, and axon components. For our electrical IC models, we will follow a similar block diagram for the basic components, including efficient models of synapses, channel regions in the soma, and communication of spikes to other synapses.

A biological neuron is defined by its soma, dendrite, synapses, and axons, as seen in Figure 3. The electrical IC models will follow a similar block diagram for the basic components. Incoming axon lines form a connection through synapses to the neuron dendrite line that feeds into the soma block of the neuron. The soma block creates the dynamics/computation to send a resulting action potential, often described as an event, to its output axon connection. The dendrite is the computation region between the signal inputs from the post-synaptic computation and the soma node for the neuron. Synapses represent the connection between axon signals and the resulting dendrite of a particular neuron.

### Channel models

**Figure 4:**
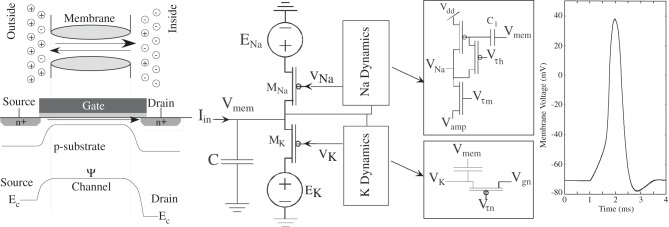
**An overview of MOSFET channel modeling of biological channels.** This approach is possible given the similar (although not identical) physics between MOSFET and Biological channels both modulated by a gating potential. The physical structure of a biological channel consists of an insulating phospholipid bilayer and a protein which stretches across the barrier. The protein is the channel in this case. The physical structure of a MOSFET consists of polysilicon, silicon dioxide, and doped n-type silicon. A channel is formed between the source and the drain. The band diagram of silicon has a similar shape to the classical model of membrane permeability proposed by Hille (2001). This approach yields an updated model for modeling biological channels that also empowers dense MOSFET implementation of these approaches. The primary design constraint is modeling the gating function with other transistor devices; such an approach is shown to model the classic Hodgkin–Huxley squid axon data, resulting in a close model to the action potential, as well as voltage clamp experiments.

The base components are based on transistor channel models of biological channel populations (Farquhar and Hasler, 2005); summarized briefly here are the key concepts as well as in Figure 4. The physical principles governing ion flow in biological neurons share interesting similarities to electron flow through MOSFET channels, and exploiting these similarities results in dense circuits that effectively model biological soma behavior. The energy band diagram (source to drain) looking through the channel of the MOSFET is similar to the energy band diagram (inside to outside) looking through a biological channel. Because the similarities between biological and silicon channels are utilized, the voltage difference between the channel resting potentials on the silicon implementation is similar to the biological power supplies. The resulting spiking action-potential circuit requires six transistors, which is the same number of transistors and just a few more capacitors (transistor size capacitors) than the basic integrate and fire neuron approach (Mead, 1989).

Other approaches are still being considered for implementing channel models (Indiveri et al., 2011), typically in systems where only the soma compartment is considered relevant (dendrite is approximated as a wire). This includes approaches implementing a range of integrate and fire neurons, including modifications to enable second order dynamics (Izhikevich, 2003), as well as models that attempt to implement some part or all of the classic Hodgkin–Huxley type channel equations (Mahowald and Douglas, 1991; Yu and Cauwenberghs, 2010). Also, other approaches have been recently considered in transistor channel modeling (Hynna and Boahen, 2006), although these approaches require more complicated circuitry without improving the channel's dynamical properties.

Solutions of ordinary differential equations (ODEs) remains an area that analog techniques are significantly more efficient than digital techniques, but given the ability to rapidly try out algorithms, digital solutions continue to be popular with a wide community. Further, there is a significant community of computational neuroscientists porting neural models to FPGAs (Cassidy and Andreou, 2009) and GPU systems, potentially resulting in leverage points. Most large-scale digital models remain to be integrate and fire networks (Izhikevich, 2003; Cassidy et al., 2007; Indiveri et al., 2011), attributing to the significant ease of such implementations over ODE solutions of channel populations (Izhikevich, 2003). The question of whether integrate and fire neurons is the correct zero_th_ order computation is still an open question.

### Synapse models

Synapses represent the connection between axon signals and the resulting dendrite of a particular neuron. The connection starts as an electrical event arriving into the presynaptic cell, releasing chemicals that reach and modulate the channels at the postsynaptic cell, resulting in a response in the dendritic structure. A Post-Synaptic Potential (PSP) is modeled typically as (Koch, 1998)

(1) $$I_{\text{syn}}=te^{-t/\tau_{\text{fall}}}$$

where τ_fall_ is typically on the order of 0.5–2 ms.

Biological synapses adapt to their environment of event inputs and outputs, where typical programming rules include long-term potentiation (LTP), long-term depression (LTD), and spike-time-dependent plasticity (STDP). In biology, synapses strengthen through chemical and morphological changes that improve signal transduction from the presynaptic to the postsynaptic cell (Markram et al., 1997; Bi and Poo, 1998).

This single transistor learning synapse has a triangle waveform modeling the presynaptic computation, a MOSFET transistor modeling the postsynaptic channel behavior, and a floating-gate to model the strength of the resulting connection. A floating-gate device is employed that can be used to store a weight in a non-volatile manner, compute a biological excitatory post-synaptic potential (EPSP), and demonstrate biological learning rules (Hasler et al., 1995; Gordon et al., 2004; Ramakrishnan et al., 2011). A MOSFET transistor in subthreshold has an exponential relationship between gate voltage and channel current; therefore to get the resulting gate voltage to get the desired synapse current, we take a log of Equation (1) to get the gate voltage, which has the shape of a triangle waveform.

**Figure 5:**
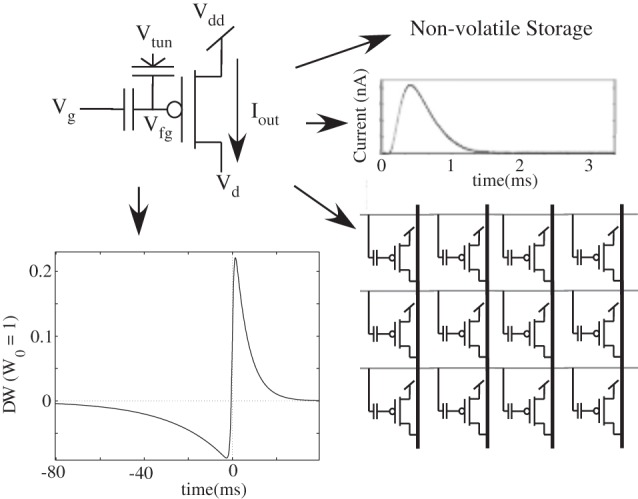
**A single transistor synapse device is presented and architecture that uses non-volatile storage, generates biological post-synaptic potential (PSP) outputs, can easily be arrayed in a mesh architecture, and demonstrates biological synapse learning rules, such as long term potentiation (LTP), long term depression (LTD), and spike time dependent plasticity (STDP)**.

A single floating-gate device has enabled both the long-term storage and PSP generation (Figure 5), but also has allowed a family of LTP, LTD, and STDP type learning approaches through the same device (Ramakrishnan et al., 2011). In this neuron chip, we have implemented these learning algorithms as part of the array, and we will summarize the key aspects of the STDP learning algorithm. The weight increases when the postsynaptic spikes follow the presynaptic spikes and decreases when the order is reversed. The learning circuitry is again placed at the edges of the array at the end of the rows, included in the soma blocks, therefore not limiting the area of the synaptic matrix/interconnection fabric. This approach has been extended to inhibitory and N-methyl-D-aspartic acid (NMDA) synapses at similar array densities. Using the transistor channel type modeling, these synapses model the current source and conductance synapse, still using a single transistor for the channel element.

**Figure 6:**
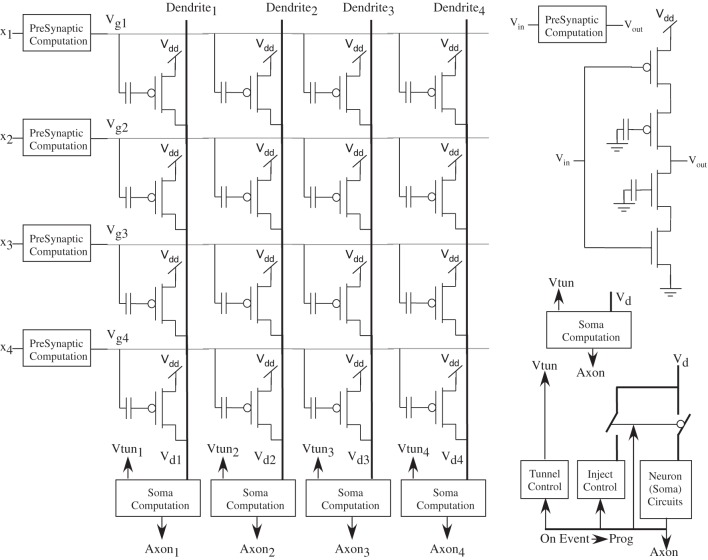
**An array of floating-gate synapses capable of adaptively modifying their weight values as well as computation and weight storage.** A dense array of synapses can be programmed, using only one transistor per cell, as in EEPROM approaches, while adding somewhat more complex circuitry on the periphery.

**Figure 7:**
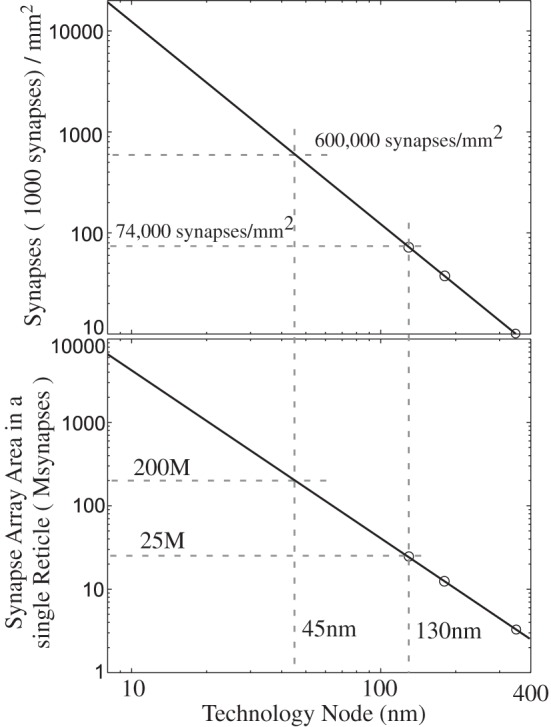
**Single-Transistor Learning Synapse density with process node.** We plot both the synaptic density per mm^2^ as well as total number of synapses per reticle, as well as data points from experimentally verified, floating-gate array of devices.

Figure 6 shows the circuit structure for an array of learning synapses; effectively we have a modified EEPROM array, with the associated density from such a structure. Current synaptic density already extrapolates to large number of synapses per mm^2^ using unoptimized devices, as seen in Figure 7; a range of optimization techniques as well as optimizing the use of input and tunneling capacitors gets the density near EEPROM levels. The data points are based on experimentally measured and publicly released values; additional data points for 45 nm and 65 nm ICs correspond well to current known research efforts. In a practical system communication is a significant issue for power consumption, as we will discuss in later sections, and related issues for Vector-Matrix Multiplication (VMM) (Schlottmann and Hasler, 2011), which shows that complexity scales linearly for mesh-type architectures.

Current EEPROM devices already store 4 bits (16 levels) in a single transistor of 100 × 100 nm area in 32 nm process (Li et al., 2008; Marotta et al., 2010). A good overview of EEPROM/Flash history was presented at ISSCC2012 (Harari, 2012). Recent data on EEPROM devices shows commercially announced devices at 15 nm (Hynix, IEDM) and 19 nm [Toshiba/ScanDisk (Li et al., 2012; Shibata et al., 2012) and Samsung (Lee et al., 2012)] as well as production of 32 nm devices. From the current EEPROM progress, such devices are expected to migrate to 7 and 11 nm technology nodes; therefore the risk that the industry will not commercially produce a 10 nm floating-gate device is very low.

Most nano-technology devices make comparisons to mesh type architectures. One expects a linear scaling down to 10 nm process to result in a 30 × 30 nm or smaller array pitch area, which is practically as small as any other competing technology, making floating gate arrays extremely competitive with other nanotechnology approaches. Even considering non-optimized floating-gate transistor arrays, one can already see the resulting scaling of these approaches. One expects that optimization of floating-gate devices for synaptic structures should yield an array density close to EEPROM densities.

These learning synapses have storage capabilities to enable them to retain 100 s of quantization levels (7–10 bits), limited by electron resolution, even for scaled down floating-gate devices (i.e., 10 nm process). Often there is a concern on the number of bits of resolution in neuromorphic systems, and although the question of bits of resolution remains a topic of discussion, floating gates and other types of neuromorphic storage often allow much denser storage than digital approaches. Since the densest synapse hardware implementation can achieve as many quantization levels as needed by algorithms, this concern is effectively irrelevant from a hardware perspective.

The density for a 10 nm EEPROM device acting as a synapse begs the question of whether other nanotechnologies can improve on the resulting Si synapse density. One transistor per synapse is hard to beat by any approach, particularly in scaled down Si (like 10 nm), when the synapse memory, computation, and update is contained within the EEPROM device. Most nano device technologies [i.e., memristors (Snider et al., 2011)] show considerable difficulties to get to two-dimensional arrays at a similar density level. Recently, a team from U. of Michigan announced the first functioning memristor two-dimensional (30 × 30) array built on a CMOS chip in 2012 (Kim et al., 2012), claiming applications in neuromorphic engineering, the same group has published innovative devices for digital (Jo and Lu, 2009) and analog applications (Jo et al., 2011).

Phase change memory is often considered a potential option for neuromorphic synapses, often due to initial success in such devices commercially (i.e., by Samsung (Chung et al., 2011; Choi et al., 2012), although earlier papers are also published). Micron started production of 1 Gbit memories in 2012. Even with all of the commercial development, the phase change memories are an order of magnitude larger area of flash devices at the same technology node, often due to selectivity issues due to high temperature controls needed for programming. In general, a single transistor is needed for programming, the same number of transistors for a flash device.

Even if the functionality was the same, then the question of additional cost of the technology infrastructure must be addressed. Further, the phase change methodolody puts into question all approaches that use external IC memories, since at some point, the value must be stored, and if digitally, requiring multiple cells per value. Such techniques include multiplexing synaptic memories to save locally on the resulting die area. The resulting issues we discuss in later sections on power efficiency and cost of communication makes such approaches prohibitively expensive.

### Comparison of fabricated ICs of soma and synapse arrays

**Figure 8:**
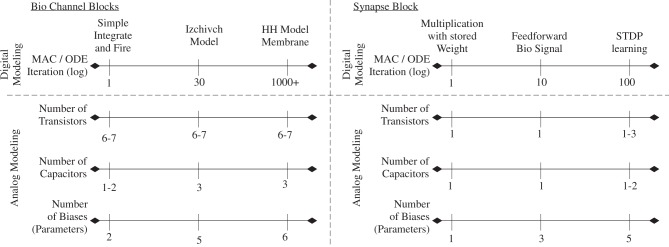
**Comparisons of implementation measures for digital and analog implementations for channel models and synapse models.** A spectrum of easy to complicated aspects understood in one area (i.e., digital) might have no similar approach in the other area (i.e., physical devices). Although the moving from an integrate and fire neuron to a HH based neuron might be a difference of 1000 in computational complexity for digital approaches, the difference in transistor, capacitor, or bias count is very small for physically implemented approaches.

Figure 8 shows a complexity comparison for channel and synaptic numerical and silicon models. Computational neuroscience community has an understanding of model complexity for digital computation based on years of research (Izhikevich, 2003). Physically based implementations do not follow the same tradeoffs, partially because we have transistor channel approaches built upon similar physics with biological devices. For example, digital computation shows a factor of 1000-fold reduced computational load when modeling with an integrate and fire neuron and HH physics based modeling (Izhikevich, 2003). For analog approaches the differences among many metrics between these two approaches is small.

Table 1 shows the structure presented in this paper results in the best synaptic density over other ICs built to date (Indiveri et al., 2006; Schemmel et al., 2006, 2008b; Camilleri et al., 2007; Brink et al., 2012). We define synapse density as the synapse area normalized by the square of the process node. Further, we achieve this synapse density in a working neural array with synapse complexity capable of high storage as well as STDP behavior; these techniques will scale down and have relatively similar density to EEPROM density at a given process node. These results demonstrate the resulting advantage of floating-gate approaches for neuromorphic engineering applications.

**Table 1:** **Comparison of synapse density and function of working implementations**.

**Chip built**	**Process node (nm)**	**Die area (mm^2^)**	**No of synapses**	**Synapse area (μm^2^)**	**Syn density**	**Synapse storage resolution and complexity**
GT neuron1d (Brink et al., 2012)	350	25	30,000	133	**1088**	>10 bit, STDP
FACETs chip (Schemmel et al., 2006, 2008b)	180	25	98,304	108	3338	4 bit register
Stanford STDP	250	10.2	21,504	238	3810	STDP, no storage
INI chip (Indiveri et al., 2006)	800	1.6	256	4495	7023	1 bit w/learning dynam
ISS + INI chip (Camilleri et al., 2007)	350	68.9	16,384	3200	26,122	2.5 w/learning dynam

*Bold value indicates synapse density as the synapse area normalized by the square of the process node*.

These approaches only consider the impact for dense simple synapses; we will discuss the impact of dendritic computation in the following areas. Having a memory that is also a transistor, as is typical for floating-gate approaches, will have advantages over other approaches.

### Dendrite models

The computation in dendritic areas is highly debated, particularly given the complexity and computational richness available here. In many modeling and implementation approaches, the dendrite is approximated to be a wire, greatly simplifying the resulting network and enabling a system that is tractable by a range of computational principles. For our discussions, the possible effectiveness of dendritic computation is considered, particularly given recent results that indicate efficient computational models using these structures.

Using channel model approaches, one can successfully build dense dendritic compartments and configurable structures (Farquhar et al., 2004) that compare well to classical models of dendrites (Nease et al., 2012). The resulting computation from dendritic elements is often debated, and in most computational models is ignored because of the increased computational complexity. Given recent results that show powerful computational aspects of dendritic structures in engineering applications (George and Hasler, 2011; George et al., 2013), it is unreasonable to ignore such effects.

### Interconnections between neurons

Communication is one of the significant differences between what would appear to be the capabilities of Si and biology. Si is mostly a two-dimensional interconnect [although there is getting to be more research efforts in limited 3D approaches (Culurciello and Andreou, 2006)] while neural tissue allows for 3D interconnection between the roughly 2D computation sheets in cortex.

Solving the 3D issue is significant for hardware implementations. On the otherhand, we can transmit events that are digital signals over wires on a digital chip in less than 1 ns; therefore it seems natural to take advantage of this aspect of the Si physics to handle event communication. Of course, to multiplex many axons on a single wire, particularly one going a long distance (over a board or sets of boards), requires a sparse firing rate among neurons. Biological neurons fire, on average, once every 2 s; this firing rate would enable such time-multiplexed communication schemes to work well, although some event coding schemes don't allow for such low event behavior.

**Figure 9:**
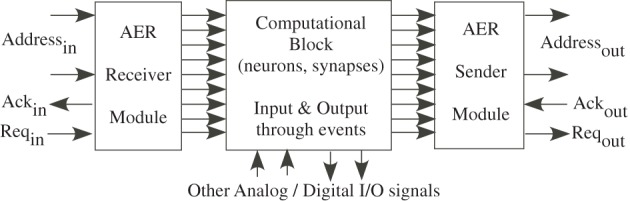
**Block diagram of our address event representation (AER) communication scheme.** AER approach is used to interface the input and output events (action potentials) of a network of neurons to the outside world. We use an AER receiver to get input events, and an AER transmitter to send output events.

The class of communication schemes that use this technique are called Address Event Representation (AER). Figure 9 shows a typical block diagram communicating events on and off the IC. For example, a typical communication is to just send an *address* from a particular neuron when it creates an event; the firing of an address communicates both that a neuron fired, and its logical address for data processing purposes. If we have a sparse number of events, then the communication happens almost instantly and without issue of collisions with other events. AER is often used to enable reconfigurability through digital storage and processing. Leaning on the digital system allows for rapid prototyping, but with significant cost in some areas (power, complexity). Current AER systems are used as a standard interface primarily between neuromorphic sensors ICs and next layer of processing connected to it. This approach enables neuromorphic systems a level of configurability and programmability using AER (and other digital interfaces) to directly communicate to digital systems.

Typical architectures could allow for senders and receiver elements in a one-dimensional or a two-dimensional scheme; a two-dimensional communication scheme often requires significant complexity in the resulting asynchronous design. One can expect a range of circuit approaches under these conditions; clearly the technique that scales with current digital design (e.g., VHDL to silicon implementation) will have a significant advantage for the entire community.

## Computation complexity toward neuromorphic application

The last section gave a sense that feasible approaches are available to build all of the basic components in digital as well as physical computational models. These thoughts lead to two additional questions:

- How do these approaches scale up to networks of neurons, say cortical neurons, of small vertebrates (i.e., fish) to mammals (i.e., mouse, cat) and finally humans?
- What computations are possible using these techniques that can compete with current implementations, whether digital or physical implementation?

Both questions are important as the computational complexity is considered that is required for neuromorphic approaches.

Although, computational neuroscience has decades of experience and significant results, finding neural system concepts that provide competitive engineering applications is only beginning. At the time of writing, the short list of particularly efficient neuromorphic computational algorithms currently proposed are

- Analog Neural Network (ANN)
- Winner-Take-All (WTA) Networks (Lazzaro et al., 1988; Indiveri et al., 2001; Chicca, 2006)
- Wordspotting (e.g., Juang and Rabiner, 1991; Lippmann and Jankowski, 1994) in groups of cortical cells

In the following paragraphs, we will model the computational load for each of these approaches as well as the computation required for a full ODE solution to the components that are currently understood. The comparisons are made in terms of the minimum digital computational complexity to perform the algorithm, and will express these comparisons in Multiply-Accumulates required for the operation. Building this framework allows for performance comparisons with traditional engineering solutions, always with an eye to where will these approaches exceed the capabilities of existing systems. Whether a digital or physical computation technique, the traditional implementation of algorithms (i.e., digital on FPGAs, DSPs, or analog on FPAAs) versus the corresponding neuromorphic implementation of algorithms are compared.

### Ann: analog neural network model

**Figure 10:**
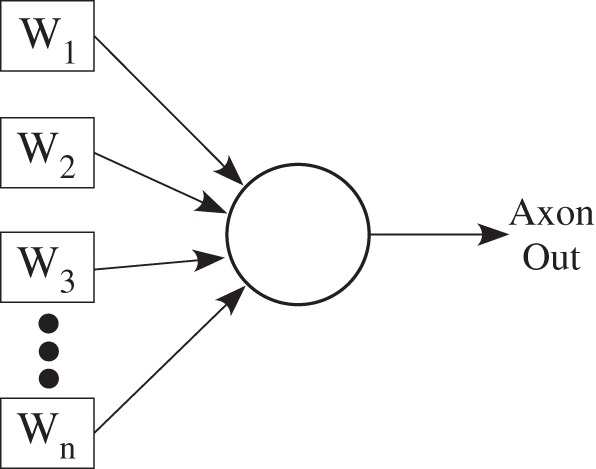
**Block diagram of a single neuron abstraction, typical in analog neural network (ANN) approaches**.

**Figure 11:**
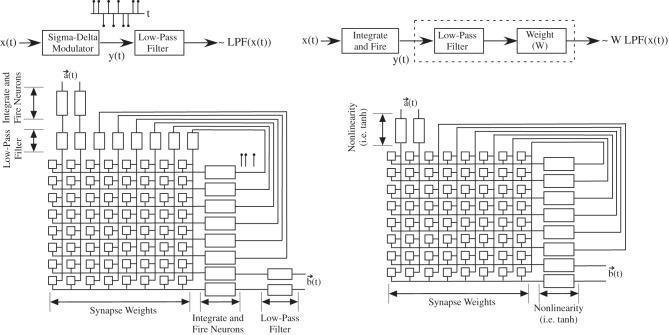
**Basic block diagram illustrating the most typical of neural models, that of a fully connected array of synapses, possibly reciprocally connected, connected to an array of neurons.** The block diagram on the left is a typical approach for an array of spiking neurons and biologically modeled synapses. The block diagram on the right is a typical approach for an array of neurons with continuous valued outputs; such an approach is called an analog neural network (ANN). The connection between spiking networks and ANN approaches starts with the realization that many neuron models, such as the family of integrate and fire neurons, are effectively linear or weakly non-linear sigma-delta modulators. Typically such functions are used for analog to digital converters (ADC), where the signal (or a low-pass filtered version) is recovered by performing a low-pass filter operation. For neural modeling, synapses effectively perform a low-pass filter on the resulting input event stream, particularly for rate encoded outputs. This model breaks down for low event rates, particularly for place coding; this case is rare for such networks which are based upon integrate and fire based neurons that are resulting in rate encoded signals. Typically, dealing with continuous-valued elements has similar implementation complexity and lower power consumption; The primary operation in either case is a Vector-matrix multiplication (VMM), with similar complexity in either case. One might find particular niche applications where one structure can be optimized over the other approach. The non-linearity block for the ANN approach might be a time-dependant non-linearity; for Hodgkin–Huxley type neurons, the resulting function resembles more of a bandpass filter function. The mesh architecture approach enables direct computing through memory.

The rise of the neural network community in the 1980's solidified a framework of neuron models that have shown a range of diversity to solve problems in many applications, so much so, that many of these techniques are considered standard techniques taught in most universities. The approach has its early roots in the perceptron (Rosenblatt, 1958) and adaptive filter (Widrow and Hoff, 1960) models that then extend to multilevel neural network models, hopfield models, as well as other related computational models. The simplest one-node approach is seen in Figure 10, where we have an input being multiplied by a weight value, all of those values added together at the soma compartment, where a linear or non-linear function is applied before we receive the output. ANN approaches include having continuous valued (i.e., tanh functions) or spiking (i.e., integrate and fire neurons, rate encoded signals) devices as well as feedforward or feedback stages (Figure 11). Often, when adding many values together, we will draw all the lines connected together and use Kirchoff's current law (sum of currents into a node equal sum of currents leaving a node) to do the summation of values; effectively this model assumes the dendrite is a wire and it performs no effective computation.

In terms of computational level, a one layer ANN would simply require the computation for the vector-matrix multiplication. Assuming we have *m* synapses (or inputs) per neuron, and *n* neurons, a complexity of *mn* would result for the synaptic computation. The rest of the computation depends on the complexity of the resulting neuron. Taking the simplest typical model, the output node would be a tanh(·) function, or roughly 4 multiply-accumulates (MAC) per neuron computation. Usually, the computation in the somas is much smaller than the computation in the synapses when *m* is of moderate size.

Figure 10 shows graphically the similarity of a spiking network of integrate and fire neurons to continuous-valued approaches. Spiking networks, rate encoded, etc., with PSP from synapses, give exactly the same computation. When a spiking network is operating with low spike rates (e.g., 1 Hz), typically seen with real neurons (with dendritic components), the computation takes a different form. At low (1 Hz and below, rare for rate-encoding) rates we probably have outputs from strong-inhibition WTA circuits (or multiple layers), and most likely an event based coding based on the location of the neuron element. Such computational approaches are open questions, although some initial applications are starting to be presented such as in robotic path-planning (Koziol et al., 2012) and sparse image/data reconstruction (Shapero and Hasler, 2012). Further, we can extend classic ANN approaches to Gaussian mixture Models (GMM), radial basis function, and other similar network approaches by taking the difference of two sigmoids.

### Winner take all (WTA) + vector matrix multiply (VMM)

WTA networks of neurons was an early area where Si engineering and neuroscience positively interacted with each other, providing a unique and efficient means of computation. As a simple definition, the network is composed of multiple (n) excitatory somas that all synapse or connect (excitatory synapses) onto a single neuron that provides inhibitory feedback connection to all of the original soma elements. The net effect is that we have an adaptive threshold, which can be global or local, that is the largest of some function on the inputs. Whether these “somas” are continuous valued or spike representations is dependent on the design and computing environment. The classic circuit implementation was based on continuous valued elements, that closely utilized transistor device physics to build an efficient circuit (Lazzaro et al., 1988). Following that success, others built multiple spike-based representations to complete the connection between these circuit approaches and biological computation (Indiveri et al., 2001; Bartolozzi and Indiveri, 2007). Further, by having local reciprocal inhibitory connections, one can make the WTA network a locally winning network, similar to WTA networks with horizontal diffusor connections between neighbor neurons. The network performs one form of an analog *max* function, which enables analog sorting computations.

**Figure 12:**
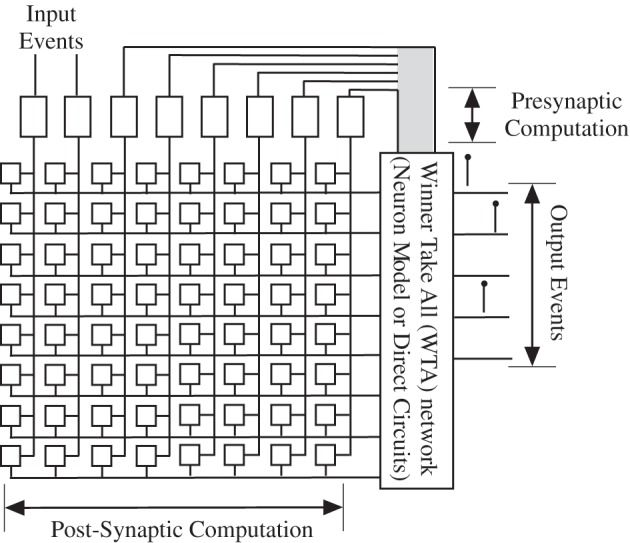
**Modeling a group of neurons using a Winner-take-all (WTA) network, along with the synaptic weighting through a typical VMM approach.** WTA has inspiration from the interactions of cortical somas and coupled inhibitory interneurons. These approaches allow for a small number of winning neurons, and sharpens up the neuron responses as well as reduces the overall spike rate. The computational power of this approach, whether using spiking neurons or as circuit implementation using a range of dynamics is still an active area of research.

The approach provides a much more accurate model of cortical computation with ANN type models; the added complexity is only at the soma compartments. Figure 12 shows the block diagram of this approach. For n somas, we have n + 1 dynamical equations. Spiking or non-spiking is similar. For effective digital numerical computation, at least a factor of 10 greater than the input samples would be needed for the dynamics. Some non-linearities are needed on each neuron to reduce their input, which in the simplest case would be say 2 MAC/element. So we are looking at approximately 30 MAC * (*n* + 1) for a WTA network for a basic structure. When we consider more local winning approaches, which are necessary, then these values clearly increase. When putting these elements into a network, one would still want a VMM at the input to model the synaptic inputs into these soma elements. As in the ANN case, the computational complexity of the synapses would be much larger than the soma elements, even for the WTA components, if *m* is large.

### Wordspotting networks

**Figure 13:**
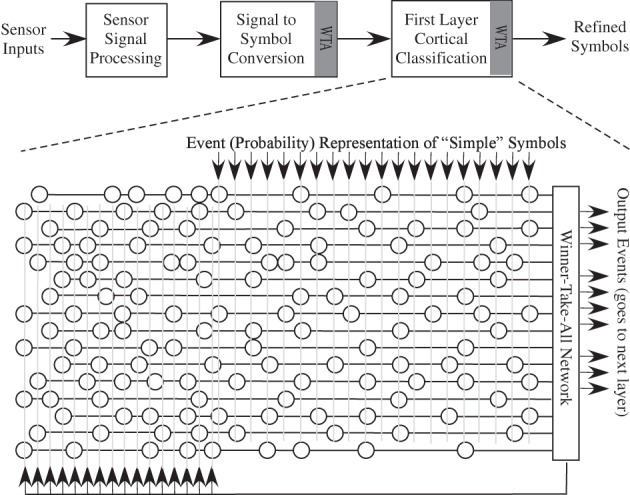
**Block diagram for computation through a number of neurons with dendritic structure; this structure comes from modeling of groups of cortical neurons.** This approach is similar to wordspotting networks used for speech recognition systems. The inputs come from another event generation layer, whether a layer of cortical neurons or as part an initial transform from sensor inputs to symbol event representation. Linear dendritic lines are being assumed in this figure, although both biological neurons and Si implementations utilize a multi-branching tree.

One recent addition to these computing platforms is a recent algorithm demonstrating experimentally in Si that neurons with at least basic dendritic structure can compute wordspotting algorithm (George and Hasler, 2011; George et al., 2013), a key engineering approach for many classifier applications. Figure 13 summarizes this approach. There are similarities between the dendritic structure and typical HMM classification structures used in speech recognition for wordspotting algorithms (Lazzaro et al., 1997), but with far more states in dendritic structures than can be practically used in any classifier.

Given this algorithm potential, we will discuss the computational complexity of this approach based on the equivalent simple HMM classifier computation; certainly both practical HMM algorithms as well as real dendritic computation is more complex. A lower bound on this computation would be 2 MAC per state variable for the required sample rate for continuous inputs. A typical dendrite would have over 1000 state variable equivalents in its continuous structure.

For a particular neuron timeconstant, τ, we would want to have multiple samples for proper operation. This discussion uses an effective discrete time sample rate 5 times more than τ; we use τ = 1 ms here. Therefore, conservatively, we have each tree computing 10 MMAC just for feedforward HMM computation. Then on top of that would be computations for learning and other functions.

The need for dendritic models is still debated in computational neuroscience (Hausser and Mel, 2003; Gonzales et al., 2011), including the resulting functionality being multiple spatially constrained neurons or more advanced features (Polsky et al., 2004); the question partially gets answered by the resulting computational efficiency demonstrated through Si based models.

The question of model detail is a classic one in computational neuroscience often debates the clear tradeoff between model complexity and computation complexity (i.e., Izhikevich, 2003), based on digital computation of ODEs. When considering electrical (and some ion related) modeling using physical (analog) hardware, transistor channel modeling, pioneered by Farquhar and Hasler, 2005, changes these constraints. For example, modeling a Hodgkin–Huxley (HH) model neuron requires 6–7 transistors directly modeling the channel population and gating mechanisms. Implementing the simplest integrate and fire neuron requires 6–7 transistors for its operation (Mead, 1989). The most effective electrical model, tuned to biological parameters like channel currents, often becomes the model of choice.

Dendritic processing is capable of significantly improved power efficiency, operating on a problem set that is well known in engineering applications (i.e., HMM, Viterbi, and related classification algorithms). Dendritic elements are a primary and fundamental structure in cortex, having a significant (factor of 1000 or better) power efficiency. Therefore, modeling a dendrite as a wire leaves far too much potential efficiency on the table. Further, such techniques would be utilized for engineering applications requiring these functions. The known efficiencies discussed so far do not make up the computational efficiency gap observed between current computers and neurobiological systems; it is suspected that neurobiological systems are computing additional functions not currently modeled.

The precision required for such operation is typically a function of system SNR, which is a function of effective capacitance (addressed in later sections) and parameter programming precision. Biological systems would follow similar noise levels, or potentially higher due to additional devices at a node, as a result of physical noise processes. Mismatch in analog is classically a significant question, a problem that is directly addressed by using floating-gate approaches; without programming approaches, these mismatch issues easily overwhelm a system design. Floating-gate elements can be programmed to 100 uV or smaller floating-gate voltage resolution, allowing precision better than 1% accuracy, better than it is believed neurobiological systems currently employ; straightforward tradeoffs are possible (i.e., increased area) if more accuracy is needed for programming.

### Full compartment ODE modeling

Another bound to the problem is provided, where we numerically compute the equivalent Ordinary Differential Equations (ODE) for each soma, dendrite, and synapse elements.

If we use a fixed sample rate, which is easier for comparison rather than adaptive rates as well as for real-time interactions, typically one uses a factor of 10 larger sample rate than the incoming signals. To numerically solve the ODE at this sample rate, we will chose a 4th order Runga–Kutta method; we would estimate roughly 10 MAC per computation, which models a few non-linearities. One can choose a wide range of methods and oversampling but generally will get similar results. Finally, for a typical line, we will assume we would need at least five state variables per node; therefore, the overhead for a single node is a factor of 500 MACs/node.

This level of computation is over 40 times larger than for the wordspotting approach. This ODE solution probably captures a better sense of the real biological computational requirements. For example, normalization and pruning of data in a wordspotting HMM classifier type model requires more computation that could be modeled by biological channel models.

### Computational model comparisons

**Figure 14:**
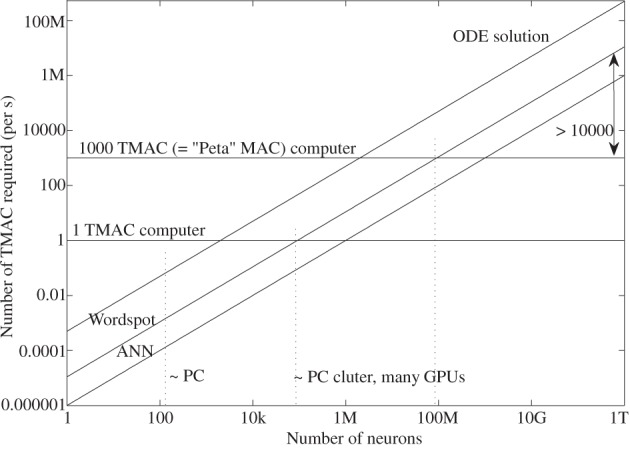
**Plot illustrating the computation described in Table 2 for the computation assuming 1000 synapses and assuming real time operating frequency (1 kHz).** One curve for the ANN and WTA complexity is plotted because they are effectively identical on this plot. To reach a computational level of 10^12^ neurons with 1000 synapses or 10^11^ neurons with 10,000 synapses, we would be missing a gap of 10,000 in the resulting computational complexity to the wordspotting approach, for the limited level of modeling of biological computation would achieve.

Table 2 shows a summary measure of the algorithms mentioned in the previous sections. These initial measure of complexity gives a sense of what is possible with classical digital computation techniques, as well as looking at comparisons for more physically based approaches. Figure 14 illustrates these tradeoffs assuming neurons with 1000 synapses each and an effective input signal frequency of 1 kHz. As we go to higher than real time speeds, we would face linearly higher more MACs per operation. All of the functions scale similarly in number of computations, but with significantly different scale factors. All lines do not include any overhead of the processor, data communication, and memory access, but rather only is there enough raw computation for the task.

**Table 2:** **Multiply accumulates per second (MACs) required for a network with m synapses per neuron and n neurons**.

**Computation**	**MAC (1 neuron/input)**	**MAC (n neurons)**
ANN	4 + m	n (4 + m) f
WTA + synapses	30 + m	n (30 + m) f
Wordspotting	30 + 11 m	n (30 + 11 m) f
ODE dendrite sim	500 m	n (500 m) f

*Other issues are assumed to be negligable for this table. Input data rate assumed to be f (Hz). Assume average dendrite has 1 compartment per synapse*.

The algorithmic comparisons between the best formulation of these particular algorithms are made to achieve the resulting functionality for digital computation. If one was to make a comparison to say a SPICE level simulation, the numbers would be significantly higher; even if we computed the resulting ODE models the computation time would be much larger than we illustrate, such as in the ANN case, or the wordspotting case, where in both cases we compare against the best case practice algorithm for that solution as the metric for the number of MAC elements. The computation to compute the resulting ODE is shown because it might be the case that the biological system is enabling that level of computation through the electrical modeling.

The incoming data rate (say 1 event per 2 s) has little to do with whether systems of neurons would need to be computed using ODEs. The total input firing rate from all synapses (i.e., 100 synapses), not the output firing rate, would directly impact an ODE sample rate. Further, given that the ODEs are multitimescale processes, resulting in stiff ODEs, the resulting digital step size for computation may be rather small even for 0.5 Hz output event rates.

Using the largest computer currently available [IBM Sequoia, 2–8 PMAC (/s) range sustained (TOP500 List, 2012)] one could build a 10^9^ to 10^10^ neuron (with 1000 synapses) ANN network, build a 10^8^ neuron wordspotting model, or build a 10^6^ to 10^7^ neuron ODE model operating in real time. For the wordspotting model, that still leaves us with 10^4^ factor in computation from a human cortex 10^12^ neurons with 1000 synapses or 10^11^ neurons with 10,000 synapses, with questions how we might achieve that resulting large hurdle. Even with a factor of 10 over current digital supercomputer architectures, we still stand far away from building a human cortex.

Physically based computation approaches give some perspective on how to approach this issue. One key aspect of physical computation, originally discussed by Mead (Mead, 1990), is that it could be a factor of 100–1000 more dense than custom digital approaches. The fundamental argument is the number of transistors that are needed for an operation is significantly less than for a digital computation, say for a multiplication. In practice, analog transistors might be slightly larger and the routing needs to be more careful than for a digital system, so in practice an efficiency improvement of 100 seems realistic. On the otherhand, most architectures have memory locally configured, reducing both complexity and memory access times, resulting in an improvement in density. In many cases, like a VMM network, there is effectively a memory array where the computation is done through the memory, and therefore, the entire computation is complete in the complexity of accessing 2–3 rows of digital memory. These modifications give promise that will enable a solution to achieving the resulting complexity for neural architectures. These approaches could reasonably be extended to other supercomputing problems.

**Figure 15:**
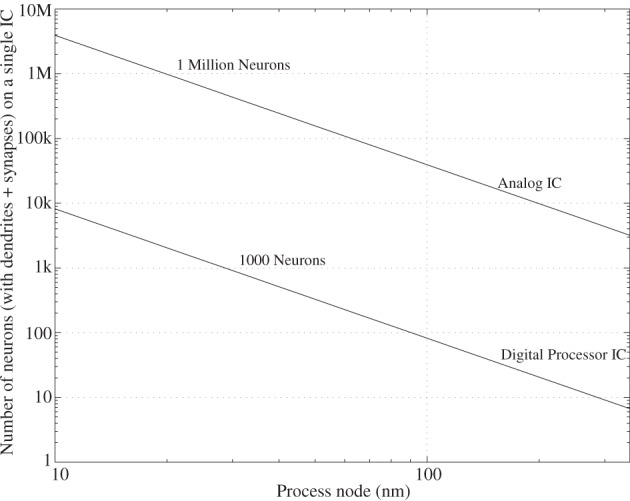
**Projection showing the number of neuron computations possible on a single IC (2 × 2 cm), assuming both digital and analog computation, as a function of process node**.

To illustrate the different complexity of computation, we will consider the relative size of digital processor as well as more physical implementations. Figure 15 shows the resulting comparison between these approaches, as well as a relative factor of 500 expected between the two approaches. Using current chip data for these approaches, it is assumed that we can implement roughly 8 pyramidal cell neurons/mm^2^ in a 350 nm CMOS process, a chip which includes local FPGA style routing as well as synaptic and dendritic modeling using local memory elements. From this data, the scaling can be approximated as roughly quadratic with process dimensions. This data per mm^2^, as well as the maximum IC size on a wafer can be plotted, typically 2 cm × 2 cm in area, or the size of the reticle stepping. Further, these approaches can be compared to an array of digital processors, typical of video processor ICs, and optimistically assume quadratic scaling with technology. A recent Nvidia IC achieves 512 processors on a single, reticle sized IC in a 40 nm process (Daly, 2011); it is assumed one processor could handle a wordspotting complexity neuron model in real time. Such an approach requires that the communication scales effectively as the resulting system is built; that issue will be discussed further when considering power dissipation issues.

What is the physical size of an analog system scaled up to human cortical levels (10^11^) neurons and 10,000 synapses? Conservatively 3 million neurons per IC would require 300,000 chips; the digital solution requires roughly 10–20 million ICs. In terms of building a physical system, one could vertically stack multiple chips in a package (e.g., 30 is possible), and one could put multiple chips on an IC board (say 100 on a 30 × 30 cm board). The analog approach requires a set of 100 boards for this architecture, which seems possible given current technologies. A similar digital system would require 40 k boards, if possible; effectively, digital solution will have a hard time reaching the complexity of a human brain, as well as having a portable application at the complexity of a mouse (i.e., 400 boards). This size system is probably not a portable system, but possible as a small rack computer.

### Silicon die cost scaling for neuromorphic computing

**Figure 16:**
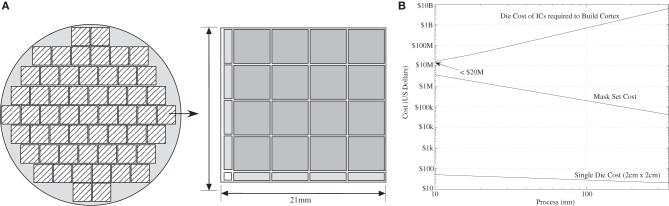
**Estimating of cost to build neuromorphic systems. (A)** A picture of a wafer, reticle, and mask, and stepping to illustrate the resulting discussion. **(B)** Estimate of mask cost, die cost, and cost of building a system at the level of human cortex for a physically based computing system. The prices for the mask and die cost are approximate, publically based information; real numbers are typically proprietary information of the particular vendor. We assume that one mask set is required for the cost of the system; in mass production of such units, the mask cost would already be spent. The resulting system cost is then almost entirely dependent on the die cost.

Silicon die area is linearly related to larger IC cost; therefore, an idea of the resulting cost of these neuromorphic type approaches is formed. The total cost for a fabricated IC is the wafer cost and the mask cost. The cost for the mask set is a one-time cost, and typically much larger than the per wafer cost. A wafer has a number of square (or rectangular) reticles that are repeated over an entire wafer; for a typical 20 cm diameter wafer, one approximately gets 50 reticles of roughly 2 × 2 cm size. Figure 16 shows a typical scaling of wafer cost with process node; a human cortex solution is entirely a question of per die cost, and would be the production cost of these system ICs. For a 10 nm IC process, the die cost would be approximately $20 M, which is high for individual households, but in the range for large commercial systems. A digital system requires a factor of 400 more ICs, so base cost would be a similar factor to these analog estimates. These costs only consider the IC cost, not the rest of the system communication and memory complexity, which will be higher for the digital computation system.

Our calculations stop at 10 nm devices, since theoretically the MOS transistor scaling stops around 10 nm devices; of course, one should never underestimate the impact of smart individuals to further push these limits, with the resulting benefits. Further, there is a possibility of new technologies pushing these limits further. To date, no technology has shown enough promise to compete with Si approaches with appropriate memory technologies. Any approach needs to compete with Si 10 nm node, the aspects of interfacing to a Si substrate, which would be necessary for any novel technology in the short term. If a technology can not show to get at least densities greater than a factor of two over a 10 nm process, the odds of its adoption is unlikely given the rest of the system complexity required.

## Power-efficiency of neuromorphic solutions

The obvious question missing after addressing the potential computational approaches, both for physical and digital processing systems, is the need to address the resulting power consumed by each system, as well as address the related question of the required communication to perform these computations. Further from Mead (Mead, 1990), it is expected that physical computing systems would be more power efficient by using physical computation techniques, and not just more area efficient computation, because of the far fewer devices needed for a single computation.

One of the amazing thing about the human brain is its ability to perform tasks beyond current supercomputers using roughly 20 W of average power, a level smaller than most individual computer microprocessor chips. A single neuron emulation can tax a high performance processor; given there is 10^12^ neurons operating at 20 W, each neuron consumes 20 pW average power. Assuming a neuron is conservatively performing the wordspotting computation (1000 synapses), 100,000 PMAC (PMAC = “Peta” MAC = 10^15^ MAC/s) would be required to duplicate the neural structure. A higher computational efficiency due to active dendritic line channels is expected as well as additional computation due to learning. The efficiency of a single neuron would be 5000 PMAC/W (or 5 TMAC/μW). A similar efficiency for 10^11^ neurons and 10,000 synapses is expected.

Building neuromorphic hardware requires that technology must scale from current levels given constraints of power, area, and cost: all issues typical in industrial and defense applications; if hardware technology does not scale as other available technologies, as well as takes advantage of the capabilities of IC technology that are currently visible, it will not be successful.

### Power efficiency in traditional digital computation

**Figure 17:**
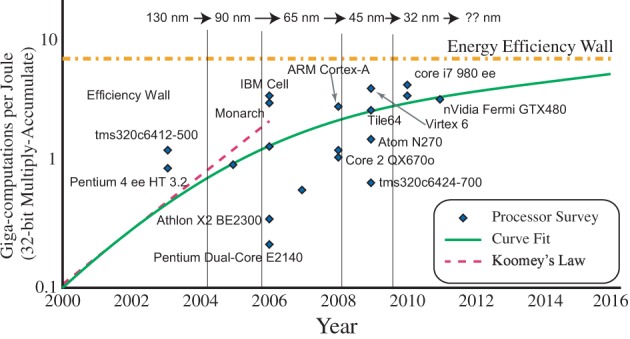
**Plots of computational efficiency for digital multiply accumulate (MAC) operations normalized to 32 bit size computation.** Over the last several years, the computational efficiency has not scaled along the lines expected by traditional Moore's law type scaling. A closer look suggests an asymptote is being reached in MAC computational efficiency using classical digital techniques. The computation efficiency levels off below 10 MMAC/mW (or 10 GMAC/W or 100 pJ per MAC). The asymptotic curve falls off from the linear trend at approximately the 90–65 nm minimum feature size node. One hypothesized factor might be mismatch between digital components requiring larger transistors, and requiring larger capacitance to be charged for each operation.

Although one might expect that conventional digital systems are simply going to keep scaling, to the contrary it certainly seems that MOSFET devices will scale to some lower limit around the 10 nm level (or smaller), and digital system performance improvements due strictly to classical MOSFET transistor scaling can no longer be expected. For example, computational efficiency of floating-point MAC units has only slowly improved over the last 11 years (factor of 2); the result is digital computation is moving toward lower precision type computations, favoring competition with neuromorphic and analog systems. Figure 17 was generated by normalizing a “computation” as a 32-bit multiply accumulate (MAC) operation (Marr et al., 2012); the approach seems independent of the particular computation architecture (DSP, FPGA, etc.); typically DSP or low-power microprocessors are used in low-power computation, due to the high baseline current required for FPGA devices (≈1 W for large devices). MAC operations are often the key aspect for high performance, signal processing, and power efficient computing, as well as is a well defined computation operation to compare approaches.

This power efficiency asymptote changes the paradigm in digital processing; one can not use single- or double-precision arithmetic without considering its cost in power. In practice, energy efficient computing systems are increasingly being designed with smaller and smaller word lengths for a particular operation to reduce the required power for the resulting computations. Decreasing the word length roughly gives a quadratic decrease in power dissipation; a limit of 100 W/TMAC for 32-bit MAC units is expected, which scales to 6 W/TMAC for 8-bit MAC computation. At 8 bit operations, conventional numerical analysis of ODEs is highly error prone and unstable, so successful use of these calculations requires reformulating models, if possible, for the dynamics. ODE computations of multiple timescales, such as adaptive filters, require significantly higher resolution to achieve reasonable SNR levels; the ideal summation in analog approaches eliminates many of these constraints. Adding non-linear operations introduces additional complexities, both in terms of MAC operations as well as resulting dynamics. Finite word length effects are still serious issues in these cases, particularly where one gets accumulation of values over a period of time. Further, expertise in small word length digital computations is rare, nearly as rare as experienced analog IC designers. Finally, at 8 bit accurate computations, the argument that digital is more accurate than analog computations is no longer valid.

One can expect innovation to improve this approach. One example of a recent asynchronous approach optimizes based on average delay rather than optimizing on worst case delay, and therefore shows results that could get past the 100 pJ per MAC barrier (Marr et al., 2012). Another approach is to consider the asymptote seems set by device mismatch; therefore, the use of programmable analog techniques (Degnan et al., 2005) might be able to overcome some of these issues. Any more specialized solution for getting past the digital efficiency asymptote requires an approach that can be pulled through the typical deep digital-design tool flow.

### Computational efficiency comparison between digital, physical, and neurobiological systems

**Figure 18:**
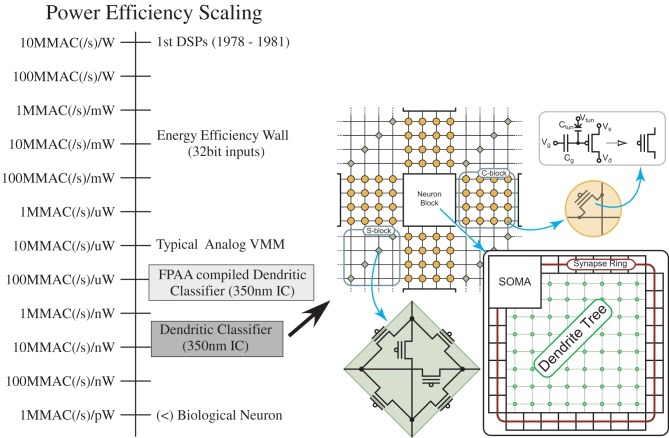
**Dendritic computation results in computational efficiency improvements over analog SP techniques.** The first approach was a compiled FPAA design, showing an order of magnitude increase, with the second, more optimized configurable approach potentially enabling three orders of magnitude over analog SP techniques (Ramakrishnan et al., 2012). The second approach was based on a local, configurable architecture (FPGA/FPAA) for routing neurons with a high percentage of local connectivity.

Figure 18 shows a viewpoint to compare ranges of power efficiencies. In this section we discuss the computational aspect of these comparisons, comparisons not including the cost of computation (communication power is zero). The next subsection looks at the cost of communication, which must be minimized to not cancel out improvements in computational power efficiency. We will consider computational efficiency versus effective capacitance, the capacitance that an additional input is required to modulate. Typically, the computational efficiency is proportional to the resulting effective capacitance; local SNR is proportional to effective capacitance due to thermal voltage. Computational efficiency is a measure that normalizes across real-time, and faster than real-time, approaches.

We approach the discussion by reviewing computational efficiency in digital and typical analog signal processing approaches, and then focus on the opportunities seen by the wordspotting structures in comparison to biological neuron computational efficiency (5 TMAC/μW), in the next paragraphs. From the previous subsection, a digital system using 8 bit MAC arithmetic is a 3 × 10^7^ factor higher than the biological computation numbers. Analog signal processing techniques have been shown to have a factor of 1000 improvement, on average, on computational efficiency for many algorithms. If we implement the biological approach as a sequence of VMM computations and similar approaches, efficiencies of roughly 10 MMAC/μW or 10 TMAC/W would be achieved; analog VMM and similar approaches are in the 1–10 TMAC/W range. Understanding neural computation offers opportunities of significant improvement in computational efficiency (5 × 10^5^).

From the discussions and data presented so far, it is expected Neuromorphic algorithm approaches are techniques that will have higher energy efficiencies than typical analog signal processing algorithms; the improvement and impact, as well as the architecture demonstrating these efficiencies, is illustrated in Figure 18. For a dendrite implementation, such as the circuit that demonstrated the wordspotting algorithm, this neuromorphic approach has higher computational efficiency compared to classic analog signal processing techniques. This implementation gives some insight into the advantages of techniques used in cortical structures. The time constant (≈ 1 ms) is set by the conductance at each node with the capacitance (C) at each node, which, in turn, sets the bias current because the transistors near rest, V_rest_, (say 10 mV above E_*k*_) are ohmic. For the dendritic line, the effective average energy per MAC equivalent operation is

(2) $$\text{Energy}/\text{MAC}=\frac{1}{2}C(V_{\text{rest}}-E_{k})V_{dd}$$

For a VMM computation, the efficiency per operation set by total effective line capacitance (C_eff_) is (Schlottmann and Hasler, 2011)

$$\text{Energy}/\text{op}=12\pi C_{\text{eff}}U_{T}V_{dd}$$

where U_*T*_ is the thermal voltage, *kT*/*q*. The effective line capacitance is capacitance at the input line divided by amplifier loop gain driving the line. In one sense, the VMM requires getting the data to the computation in a matrix array, with the associated capacitance; with the dendrite approach, the computation starts closer to the inputs. Getting the data to that part in the computation would be a separate discussion, and is addressed in the following section.

**Figure 19:**
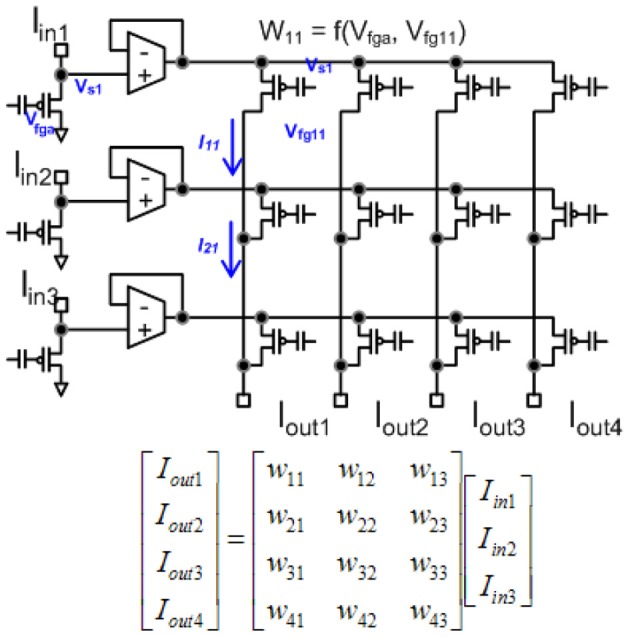
**Programmable floating-gate transistors performing a vector matrix multiply using current-domain mathematics**.

Both approaches scale linearly with power supply voltage (V_*dd*_); decreasing the supply results in a proportional improvement in efficiency. Typical numbers are mentioned for V_*dd*_ at 2.5 V. For a VMM, one could imagine decreasing the supply voltage to 0.5 V, probably limited to the driving amplifier headroom. The dendritic line, with the use of programmable analog elements, should be able to decrease the supply voltage to biological levels (180 mV) (Siwy et al., 2003). For a digital structure, the dynamic power decreases with V^2^_*dd*_ due to switching energy, and is proportional to the capacitance of the entire multiplier circuit. The capacitance of the entire multiplier element is orders of magnitude larger than a typical single floating-gate transistor doing an equivalent vector-matrix multiplication shown in Figure 19. Static digital power tends to increase with decreasing *V*_dd_ (Kim et al., 2003), and can offset the resulting gains, as well as increase transistor mismatch, requiring larger (Width * Length) devices and larger capacitance.

**Figure 20:**
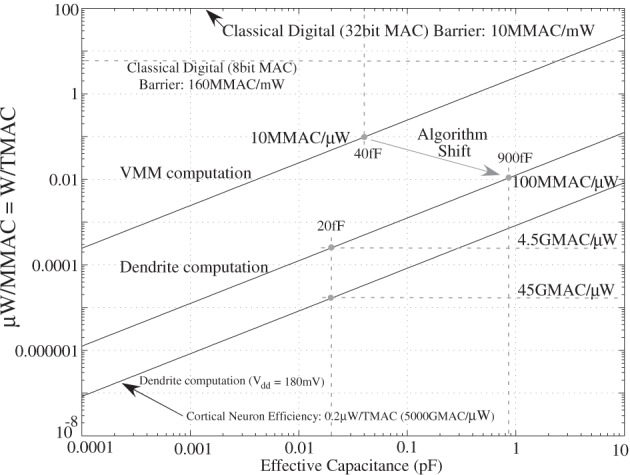
**Plot of computational efficiency versus capacitance level for VMM (analog) and Dendrite computation (neuromorphic, wordspotting) physical algorithms for V_*dd*_ = 2.5 V.** For both algorithms, the efficiency improves linearly with decrease in V_*dd*_, since power scales linearly with V_*dd*_ here. We also show the computational efficiency for the dendrite computation for V_*dd*_ = 180 mV, typical of neurobiological systems (Siwy et al., 2003). We also include a table of effective SNR, computed from thermal noise at the node over signal size (≈*U*_*T*_), as a function of capacitance.

Using the equivalent computation of a network of cortical neurons in Table 2, the different computational approaches are compared. Figure 20 plots computational efficiency versus effective capacitance, as well as providing a comparison between these computational approaches. Effective capacitance is defined as the resulting increase of charge required for an additional node of the computation occurring in parallel. The classical 32-bit MAC digital power wall is at the top of the graph, and the power wall for 8-bit computation is nearly at the top of the graph; power efficiency would scale as the total capacitance for the digital operation. When power is a constraint for a digital system, SNR can not be assumed to be effectively infinite. A typical value for a VMM compiled in an FPAA would be at 10 MMAC/μW (=10 TMAC/W) power level. By utilizing the computation efficiency in dendritic structures for wordspotting approaches, a basic compiled structure with large node capacitances (i.e., ≈ 1 pF) shows an improvement in power efficiency of a factor of 10, a more dedicated approach would show an improvement of 450 over the VMM structure. Decreasing the resulting power supply to biological levels (V_*dd*_ = 180 mV), shows another factor of 10 improvement in power efficiency (45 PMAC/W). All of these factors, with typical node capacitances results in structures within two orders of magnitude of the power efficiency of biological systems; the Si internode capacitance could be further decreased as nodes scale down. These neuromorphic techniques show promise to approach the computational efficiency and raw computational power as mammalian nervous systems.

**Table:** 

Capacitance	1 fF	10 fF	100 fF	1 pF
SNR (dB)	22.1	32.1	42.1	52.1

Further, scaling capacitance at each node has a direct impact on the thermal noise at that location, whether in a silicon or biological system. The best case (lowest level) for thermal noise current ($\hat{I}$) in a device is related to its bias current (*I*) as

(3) $${\hat{I}}^{2}/I^{2}=\frac{2q\Delta f}{I}$$

where Δ*f* is the bandwidth of interest; for a Si transistor in saturation, we exactly reach this level (Sarpeskar et al., 1993). Low current levels are often needed to achieve the resulting power efficiency, which requires programming to low currents (i.e., pA levels, similar to biological levels), leading to lower, classically measured SNR levels, typical of biological systems. For example, for 1 kHz bandwidth, we get a relative noise variance as

**Table:** 

I_bias_	10 fA	1 pA	10 pA	1 nA
% noise	20	2	0.2	0.02

Further, for coupling of capacitors with transistor source junctions (subthreshold), the noise level is related to the familiar *kT*/*C* = (*U*_*T*_/*q*)/*C* noise, where C is the capacitance at that node. Figure 20 shows a table of SNR at each of these capacitance nodes, which are consistent with the low currents mentioned above.

As capacitances scale down, the resulting bias currents for the real-time performance will also decrease as a result. For neuromorphic circuits, faster than real-time performance is not only possible, but often easier. Fortunately, MOSFET transistors can easily handle smaller currents, although for lower threshold voltage processes, either the source voltage must be moved relative to the substrate or the gate voltage must be outside the resulting power supply voltages, easily achieved with floating-gate devices. Typically, the lowest currents are bounded by the dark current in the drain and source junction devices, limiting current levels in the 1–10 fA range in practice, but still enabling biological time constants with small (say 1–10 fF) capacitances. The current levels, as well as the resulting thermal noise levels, would be similar to biological levels.

## Power efficient neuron event communication

In the previous section, we have developed models on computation scaling, particularly requirements toward cortical computing requirements. These models are necessary for understanding computation, but not sufficient because we need to consider the resulting power dissipation for communication. So for this discussion, a computational scheme that fits the power budget is assumed, as modeled in the previous section, particularly for a cortical structure. To consider the power consumption for communication, we must consider communication of events, memory access, and resulting infrastructure requirements, discussions we did not address in the previous section.

### Constraints from biological computation

For biological systems, the communication is primarily communicating events, or action potentials, which are effectively digital signals. In some cases, we might start preconditioning signals for computation, but where successful, it has minimal effect. Analog encoding is possible, and might have power efficiency improvements if the event encoding is directly representable in analog signals, which for non-rate encoded signals is challenging. For the remainder of this section, we assume we are communicating digital events between neurons.

Neurobiological computation systems also address power efficiency constraints. The human cortex consumes about 20 W of power, of which, only a fraction of this power is used for computation; going forward, we will assume 25% of average power (5 W) for communication of events from somas to synapses. One formulation for switching energy, which is commonly used in digital for charging or discharging a capacitor is

(4) $$\text{Energy}=\frac{1}{2}C_{L}V_{dd}^{2},$$

where C_*L*_ is the capacitive load, and V_*dd*_ is the power supply, which for a biological communication is between 140 and 180 mW (Hodgkin et al., 1952). The total energy for a biological event is twice this value (using the digital modeling of charging and discharging a capacitance). Calculating capacitance from power in a digital model, given a typical spike rate in the cortex occurring once every 2 s (0.5 Hz firing rate), and 10^12^ neurons in the cortex, this results in 245 pF total capacitance on an axon line for a biological system, corresponding to 30.6 mm average total cable length of 1 μm diameter axon cable (fairly thin axon). This calculation shows that digital communication must be constrained to replicate the low switching energy of the biological system. Average event rate for neurons in cortex has been consensus below 1 Hz, although that level depends on region to region of cortex [i.e., Early auditory cortex is 2.5–4 Hz average rate (Kock and Sakmann, 2009; Koulakov et al., 2009; Roxin et al., 2011)] (Sejnowski and Churchland, 1992; Kock and Sakmann, 2009). Typical axons range in diameter from 1 to 20 μm, although values outside this range are found (Verveen, 1962; Debanne et al., 2011), and typically have elaborate arborization patterns to large numbers of neurons, often within a single region of the brain (Debanne et al., 2011). Mylenation will extend the length due to lower capacitance, particularly for larger axons which also have larger diameters; small, thin axons tend to have little mylenatation axons. If a typical sum total length of all mylenated axons in the human brain is 1.5 × 10^8^ m (Kandel et al., 2000), the resulting axon length for a particular neuron is 1.5 mm increase of the 30.6 mm average cable length per neuron; the effect mostly increases the length of long-distance connections.

The net result is that with most communication on biological axon lines, even though they might be present everywhere, including intricate three-dimensional patterns, one does find an exponentially decreasing distribution of axon cable length in cortex, consistent with the neural communication being constrained to a tight power budget. This result is consistent with data that most neurons have a high level of local interconnection (Douglas and Martin, 2004), such as nearby cortical neurons; any cortical architecture must explicitly incorporate these effects to achieve the necessary power efficiency gains. Further, these results are also consistent with the low average spike rates found in cortical systems (1 spike per second); an entire cortical network operating with rate encoded signals (i.e., 3–300 Hz) would consume 100 times the power, and therefore the axon cable length for a cortical power dissipation requires 100 times shorter cables, which is impractical. We expect that constraining silicon communication power may be required based on this biological inspiration.

### Constraints from digital computation systems

Classical digital computation systems have considerable depth of experience in communication of digital signals, including event structures. For typical CMOS communication, (4) is directly relevant to digital systems communication; for source coupled approaches (Emitter or Source Coupled Logic), the V^2^_*dd*_ term is modified by voltage swing times V_*dd*_, resulting in somewhat lower dynamic power but potentially higher static current; we will focus on the classical approach through this discussion, which will have minimal differences for other encoding schemes. Classically, communication of information over a longer distance is expensive in power; a good summary for these approaches is written elsewhere (Culurciello and Andreou, 2006). The capacitance for a line is a function of the distance of the connection, as well as making connections from one package to another or making connections between boards or other approaches. Given digital communication is fast, in theory, communication could happen with small delay; a low average spike rate is essential in having the communication being nearly instantaneous.

**Figure 21:**
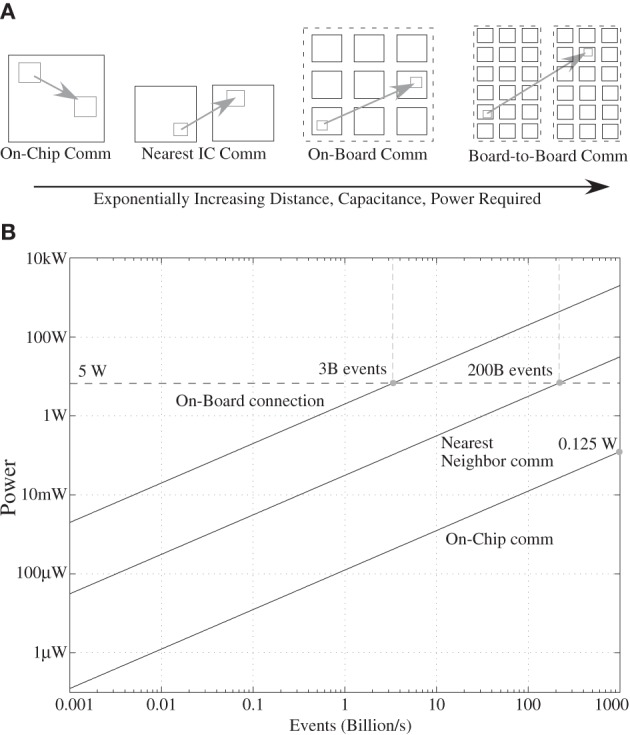
**Modeling of power required for transmitting an event. (A)** We consider computation between devices on a single IC, between neighboring ICs, on a single board, and distances beyond a single board (i.e., between two boards). Each of these steps requires considerably more power for communicating the resulting event; the more local the communication, the more power efficient the resulting computation. **(B)** Communication power versus number of events (Gbit) communicated. We consider the three cases of transmitting a bit on a chip (average C_*L*_ = 1 pF, V_*dd*_ = 0.5 V), transmitting a bit to a neighboring chip (average C_*L*_ = 10 pF, V_*dd*_ = 2.5 V), and transmitting an event address of 8 bits on a board (average C_*L*_ = 80 pF, V_*dd*_ = 2.5 V). Each case requires 0.12, 31.3 pJ, and 2 nJ energy communication per bit, respectively. We would expect even more power consumption for longer distance communication (i.e., between boards), because of the larger capacitance for these approaches. On board requires address communication, because when transmitting sparse events encoding the address gives an optimal solution.

Figure 21A shows a few representative levels for communication of events, typical boundary locations for typical communication. Where possible, we want to have as much communication locally on a single IC for low-power operation, since that decreases the total amount of capacitance needed to be charged and discharged (i.e., 1 pF for long distance connection on chip), as well as allows for a (lower) range of V_*dd*_ could be supplied as well as a range of possible communication schemes. Further, the tighter integration between memory elements and computation further decreases communication power; ideally, as in the STLS approaches, the memory and computation are integrated together, eliminating this particular issue. The types of approaches at a local level needed to optimize the use of memory in the routing architecture. For example, efficient FPGA approaches achieve both approaches, integrating the non-volatile memory for the connections with the communication of events in a low capacitance infrastructure. Further, dendritic structures bring more of the information refinement to the axon outputs.

Almost all systems require communication between multiple chips. When communicating events with a neighbor chip (e.g., 1 chip right next to the transmitting IC), the minimum capacitance is typically set by 10 pF by specification (due to packaging, bonding, etc.), as well as off chip communication tends to be at larger V_*dd*_ (5, 3.3, 2.5 V; we assume 2.5 V for these calculations), resulting in a higher energy computation. Such an approach results in 31.3 pJ per bit [or 31.3 μW/(Mbit/s)] independent of the communication scheme. Such event communication schemes could transmit an event in only a single bit on the resulting line. Further, the introduction of 3D silicon processing (die stacking, multiple grown layers, etc.) has introduced technologies that can reduce the effective off chip capacitance by an order of magnitude, and therefore, such approaches should be utilized where available in a particular technology for multichip approaches.

When we communicate over distances longer than nearest neighbor chips, we typically employ an Address Event communication scheme (i.e., AER), which requires sending the location of a particular spike between chips. At least, this requires an address for the particular line, as well as the particular chip we are considering; on a single board, an 8 bit address would be a lower limit for such approaches. In such an approach, a communication of an event would travel multiple minimum chip distances (i.e., 8 is a lower bound for an average number), resulting in roughly 2 nJ per operation. As we go to longer distances, and particularly when we go to different boards, we see a significant increase in capacitance and addressing as well as routing infrastructure; the goal is to minimize the number of such long distance events that need to be communicated, while preserving the capability.

Figure 21B shows a graph of the power required for communicating a number of events for these different schemes. When trying to reach biological efficiencies for communication, we have significant limits even communicating single events between neighboring ICs, not to mention longer distance communication. For 10^12^ events per second results in 30 W of power consumption (1 Tbit/s). The result requires most of the computation to be local; fortunately, neurobiological systems use a similar approach in the fact that over 90% of neurons in cortex project locally to nearby neurons (i.e., nearest 1000 pyramidal cells).

For example, if the off chip (not nearest neighbor communication) to is budgeted for 1 W of power, then only 0.05% of events can use this communication channel. Further, if we budget 1 W for off-board events, then with the additional capacitance and bits for selection needed, one would see 64 times more capacitance, resulting in 0.001% events communicating off board. As additional technology becomes available, such as multiple die stacking in a given package or three-dimensional circuit fabrication, the resulting capacitance for communication will decrease, improving some of these numbers, but the containing concepts will still be the same. We expect similar type issues in neurobiological systems; even though the brain can communicate over long distances by many wires, the resulting energy to do so would be prohibitive in its current energy budget. Such constraints keep the communication overhead for the system manageable, and therefore the communication structure never becomes too large a burden for the system scaling to large sizes.

The low spike rate has a similar effect for synthetic systems as it does in biological systems; increasing spike rate by a factor of 100, typically necessary for implementations using rate encoded approaches, increases power by at least a factor of 100, significantly limiting where such systems can be used. Of course, most rate encoding approaches simplify neuron elements to elementary sigma-delta converters, eliminating most of the computational possibilities.

**Figure 22:**
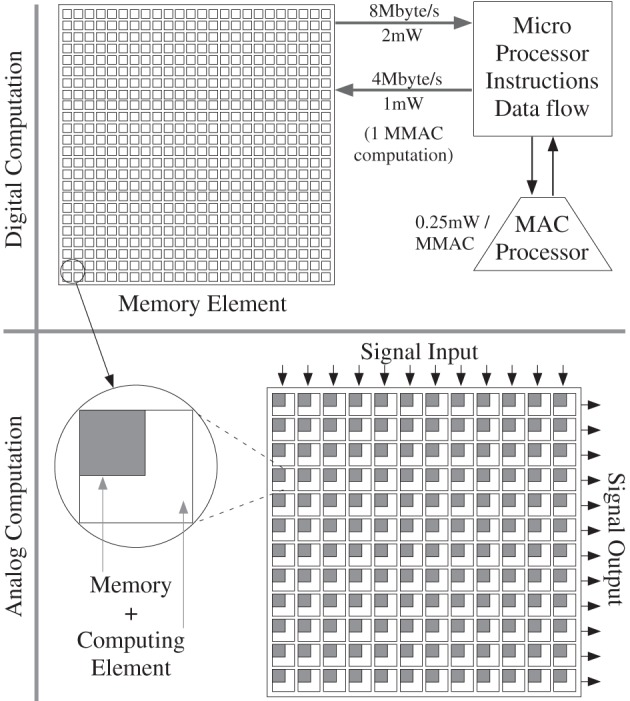
**Diagram showing typical computation models for digital and analog approaches.** For a typical digital computation, we must access the data (as well as instructions), communicate it to the processor, perform the computation, and communicate the results back to the memory. When this memory is an off-chip device, the resulting power consumed for communication is much higher than an efficient processor. The analog approach directly computes through the memory, and therefore minimizes the resulting issues and complexity due to communication. One could use digital based computation and memory to achieve some advantages, limited by the computational efficiency limits for digital techniques.

Rarely is the digital communication included in power for computation (Figure 22). For example, the computation power to access 1 MMAC of data from a nearby memory block, requiring two 2 Mbyte, 32 bit input data, and 1 Mbyte, 32 bit output data, results in 3.1 mW (V_*dd*_ = 2.5 V) of power, even though one might find a DSP chip computing at 4 MMAC(/s)/mW power efficiency (TMS320VC5416, 2008). A memory chip or data source further away requires even higher level of power. As another example, using a memory element one chip away for remapping neuron addresses, which is usually a first step to storing synaptic weights in off-chip memory, requires sending an 8 bit address off the chip and an 8 bit address back on the chip. Just this power alone requires 0.5 nJ per remapping in the best case; at 10^12^ events/s, we require 500 W for this simple computation. Such an expensive computation must be used in particular targeted areas.

**Figure 23:**
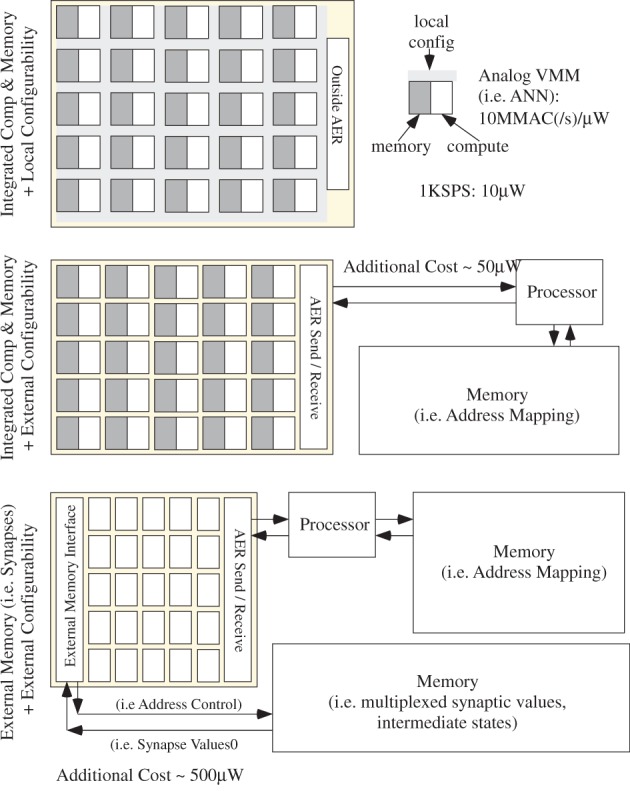
**Illustration of the costs of external communication for configurability and storage.** Where possible, we want data-flow operations where memory and computation are co-located with local routing/configurability. Moving configurability is moved off of the processing die substantially increases computational cost because of the power and complexity requirements for moving the data to an external processor/memory, even if next to the IC. Moving memory away from Processing, say for multiplexing Synaptic values, further increases the resulting power and complexity cost, even if the original device gets simplier and smaller. These schemes include rate encoded approaches encoding synapse values because of the increased event rate. We include values for a small network of 1000 neurons with 100 synapses operating with a 1 KSPS operating speed assuming a typical ANN (i.e., Vector-matrix multiplication) neuron structure.

Figure 23 shows the tradeoffs between these systems, as well as simple comparisons between a small network of simple neurons and synapses. Using external memory as the primary approach for programmability and configurability, as is the typical use of AER communication schemes, comes at a huge cost that makes scaling to large systems impractical. The advantages of AER communication, which include enabling long-range, sparse interconnections, comes with the added cost of digital communication, costs that are very small for sparse, infrequent events, and that depend on the distance required for communication (on-chip, off-chip, off-board). Adding the additional cost of FPGA or other high performance digital processing only further weakens the applicability of these approaches going forward. One sees exactly the same issue when using multiplexing of a memory with an analog system, whether to load synaptic weights in an external memory. This result shows the heavy energy cost of computation and memory that are not co-located; although this approach might have advantages in initial system building, it requires communication across sizable capacitance, and therefore requiring more power, as well as system complexity.

### Energy efficiency comparisons for other neuromorphic implementations

Many neuromorphic systems claim to be power efficient, and compared to typical digital off-the-shelf approaches, these claims are often right. In each of these approaches, the IC power efficiency is between the digital and analog SP techniques, with much lower system power efficiency due to the high-level for communication overhead (including FPGAs for routing). Many techniques start with a power efficient neuromorphic sensor, such as the DVS imager (Lichtsteiner et al., 2008), which compares well to commercial cameras, making it a favorite sensor interface for many neuromorphic platforms. Unfortunately, neuromorphic techniques have not often improved past the analog SP efficiency; often the approaches, including event-based approaches, reduce down to Vector-Matrix Multiply operations, as sometimes explicitly said by the authors (Serrano-Gotarredona et al., 2009). These facts leave us with a small list of potential neuromorphic computational models currently used; the authors believe more efficient algorithms will be discovered/invented over the coming years.

We will comment on a few representative neuromorphic systems, while amazing feats of engineering as platforms for neural simulation and modeling, do not reach the desired power efficiency targets. The Caviar project illustrated a heroic effort building large-scale neuromorphic processing capabilities using the computation from the DVS imageer (Lichtsteiner et al., 2008). The resulting convolution IC, the primary workhorse of the architecture, was capable of 12 GMAC, low-precision operations in roughly 100 mW of power; these impressive numbers are still two orders of magnitude less power efficient than VMM type operations, even though the core operations are similar. The resulting system integration cost is significantly higher (even when not using USB monitors of USB events) as well as requiring FPGA ICs for routing (i.e., synapse mapping), as a tradeoff for system modularity; lower event rates would further improve the resulting system. Related algorithms using DVS imagers, while computationally interesting including stereo processing (Ni et al., 2012; Rogister et al., 2012), show useful neuromodeling approaches considering practical algorithms, but often computed on a standard digital computer. The possible efficient implementation being better than the analog SP line is neither demonstrated theoretically or experimentally at this time.

The SpiNNaker approach (Furber and Brown, 2009; Rast et al., 2011; Furber, 2012; Painkras et al., 2012) uses efficient event-based communication structures, but utilizes 18 standard ARM968 integer-math processors (≈ 4 GIPS in 1 W) for solving any of the neuron dynamics, and therefore will be almost as efficient as the digital power-efficiency wall, far from the analog SP computation possibilities. Further power limitations occur when the processors require off-chip memory, typical of many current implementations. Other resulting systems, such as Neurogrid (Lin et al., 2006; Silver et al., 2007) and Wafer level implementations from the group centered in Heidelberg (Schemmel et al., 2008a,b) in best cases get close to the analog VMM efficiency, typical of an ANN.

Any practical neural implementation must make sure that the resulting infrastructure does not overwhelm the efficient computation. Such an implementation must consider system communication of events, communication to outside processors, and other multiplexing structures. Without architectures that can, in the particular implementation technology scale from one to billions of neurons, clearly has advantages over other approaches. Many previous attempts to scale up single or small networks of neurons have often slowed down development because of these issues. The Silicon Cortex Project (SCX, from INI) spent enormous engineering effort to communicate between a few neurons on a single board in the multi board system (Deiss et al., 1999; Indiveri et al., 1999); the Central Pattern Generator (CPG) system by Patel et al., faced similar issues (Patel et al., 1999, 2006). The resulting system design for the communication, programming, and configuration infrastructure far outweighed the neuromorphic computation issues. Even successful multilayer model implementation are constrained by similar approaches, and face significant challenges to scale past current levels, primarily due to the digital communication infrastructure (Lin et al., 2006; Silver et al., 2007; Schemmel et al., 2008b; Serrano-Gotarredona et al., 2009).

## Commercial considerations to drive these systems

Although one can discuss how to build a cortical computer on the size of mammals and humans, the question is how will the technology developed for these large systems impact commercial development. The cost for ICs alone for cortex would be approximately $20 M in current prices, which although possible for large users, would not be common to be found in individual households. Throughout the digital processor approach, commercial market opportunities have driven the progress in the field. Getting neuromorphic technology integrated into commercial environment allows us to ride this powerful economic “engine” rather than pull.

In most applications, the important commercial issues include minimization of cost, time to market, just sufficient performance for the application, power consumed, size and weight. The cost of a system built from ICs is, at a macro-level, a function of the area of those ICs, which then affects the number of ICs needed system wide, the number of components used, and the board space used. Efficiency of design tools, testing time and programming time also considerably affect system costs. Time to get an application to market is affected by the ability to reuse or quickly modify existing designs, and is reduced for a new application if existing hardware can be reconfigured, adapting to changing specifications, and a designer can utilize tools that allow rapid modifications to the design. Performance is key for any algorithm, but for a particular product, one only needs a solution to that particular problem; spending time to make the solution elegant is often a losing strategy.

The neuromorphic community has seen some early entries into commercial spaces, but we are just at the very beginning of the process. As the knowledge of neuromorphic engineering has progressed, which have included knowledge of sensor interfaces and analog signal processing, there have been those who have risen to the opportunities to commercialize these technologies. Neuromorphic research led to better understanding of sensory processing, particularly sensory systems interacting with other humans, enabling companies like Synaptics (touch pads), Foveon (CMOS color imagers), and Sonic Innovation (analog–digital hearing aids); Gilder provides a useful history of these two companies elsewhere (Gilder, 2005). From the early progress in analog signal processing we see companies like GTronix (acquired by National Semiconductor, then acquired by Texas Instruments) applying the impact of custom analog signal processing techniques and programmability toward auditory signal processing that improved sound quality requiring ultra-low power levels. Further, we see in companies like Audience there is some success from mapping the computational flow of the early stage auditory system, and implementing part of the event based auditory front-end to achieve useful results for improved voice quality. But the opportunities for the neuromorphic community are just beginning, and directly related to understanding the computational capabilities of these items. The availability of ICs that have these capabilities, whether or not one mentions they have any neuromorphic material, will further drive applications.

**Figure 24:**
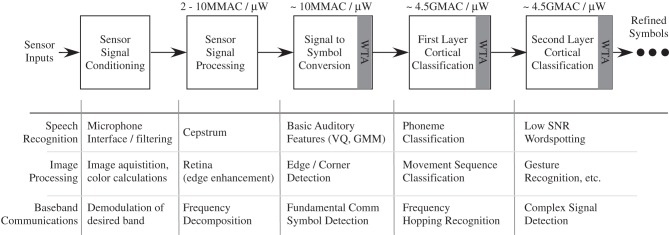
**Typical signal processing chain using configurable analog approaches and neural based classifiers.** Once the input signal becomes established as a refined probability of low-level symbols, through a WTA approach (Lazzaro et al., 1988), we have a cascade of classifier layers typical of processing in cortex.

One expects that part of a cortex processing system would have significant computational possibilities, as well as cortex structures from smaller animals, and still be able to reach price points for commercial applications. In the following discussion, we will consider the potential of cortical structures at different levels of commercial applications. Figure 24 shows one typical block diagram, algorithms at each stage, resulting power efficiency (say based on current technology), as well as potential applications of the approach. In all cases, we will be considering a single die solution, typical for a commercial product, and will minimize the resulting communication power to I/O off the chip (no power consumed due to external memories or digital processing devices). We will assume a net computational efficiency of 10 TMAC/mW, corresponding to a lower power supply (i.e., mostly 500 mV, but not 180 mV) and slightly larger load capacitances; we make these assumptions as conservative pull back from possible applications, although we expect the more aggressive targets would be reachable. We assume the external power consumed is set by 1 event/second/neuron average event-rate off chip to a nearby IC. Given the input event rate is hard to predict, we don't include that power requirement but assume it is handled by the input system. In all of these cases, getting the required computation using only digital techniques in a competitive size, weight, and especially power is hard to foresee.

We expect progress in these neuromorphic systems and that should find applications in traditional signal processing and graphics handling approaches. We will continue to have needs in computing that outpace our available computing resources, particularly at a power consumption required for a particular application. For example, the recent emphasis on cloud computing for academic/research problems shows the incredible need for larger computing resources than those directly available, or even projected to be available, for a portable computing platform (i.e., robotics). Of course a server per computing device is not a computing model that scales well. Given scaling limits on computing, both in power, area, and communication, one can expect to see more and more of these issues going forward.

We expect that a range of different ICs and systems will be built, all at different targets in the market. There are options for even larger networks, or integrating these systems with other processing elements on a chip/board. When moving to larger systems, particularly ones with 10–300 chips (3 × 10^7^ to 10^9^ neurons) or more, one can see utilization of stacking of dies, both decreasing the communication capacitance as well as board complexity. Stacking dies should roughly increase the final chip cost by the number of dies stacked.

In the following subsections, we overview general guidelines to consider when considering using neuromorphic ICs in the commercial market, first for low-cost consumer electronics, and second for a larger neuromorphic processor IC.

### Small, high-volume consumer electronics ICs

In one case, we will consider a small die of 1 mm^2^ (10 nm process node), typical of commodity parts say in audio devices or cell phones components (Table 3). The cost is roughly a linear function of the die area, but also a function of packaging, testing time, production costs, and sales cost. We might expect a chip cost of $2 range, resulting from a die cost less than $1. In 1 mm^2^ area, we could imagine a network of 60,000 cortical neurons, resulting in 10 TMAC equivalent computation in 1 mW of power. We assume roughly 1000 neurons project outside of the IC per second, therefore with addressing bits would require 4 kb/s, resulting in 125 nW of average output communication.

**Table 3:** **Table of possible specifications for commercial Neuromorphic ICs**.

	**Consumer IC**	**Processor IC**
Die size	1 mm^2^	40 mm^2^
Chip cost	$2	$100
Neurons	60,000	3,000,000
MAC	10 TMAC	500 TMAC
Comp power	1 mW	50 mW
Out events	1000/s	10,000/s
Comm power	70 nW	8 μW

Even at the price point for a high-volume commercial device ($2 range, Table 3), we have computational power rivaling most computer clusters and arrays of graphics chips integrated as a component on a board. Potential applications are as a word spotting front-end, and robust speech recognition in low SNR environments. A practical application would require some level of analog signal processing to create the input symbols for the computation, similar to the pathways we see leading up to cortex from the sensory systems. Further, these systems can be operated at frequencies higher than real time, requiring a linearly increase in power consumed for increase in operating frequency; these approaches could enable using these techniques for front-end classification of baseband communication systems.

### Potential of a neuromorphic processor IC

In another case, we will consider a large die of 400 mm^2^, the size of an entire reticle, typical of the microprocessor ICs, graphics ICs, and other higher end commercial ICs. We might expect a chip cost of $100 range, resulting from a die cost under $50 per die, given current pricing models. These chips would probably exist in handheld or other electronic devices that sell above a $350 range, which enables a wide range of commercial applications. In 40 mm^2^ area, we could imagine a network of 30,000,000 cortical neurons, resulting in 500 TMAC equivalent computation in 50 mW of power. We assume roughly 10,000 neurons project outside of the IC per second, and with addressing bits would require roughly 256 kb/s, resulting in 8 mW of average output communication power.

By comparison, these numbers show effectively a hand held device having the computational power rivaling the largest of today's supercomputers in the power consumed by less than most handheld devices, and at a price point that could be put into higher end commercial devices, such as tablets or laptops. Potential applications would include the speech recognition examples for the smaller chip, as well as (or in addition to) image processing emulation, particularly on 1 M pixel images, including receptive field processing, image/scene classification, and pre-attention mechanisms.

### Tools for designing neuromorphic systems

Modern system design expects a design environment to work through all of the layers of abstraction to achieve reasonable application performance; we should expect a similar approach for neuromorphic systems.

In many cases, we can utilize existing tools, where they exist, such as microcontroller programming or FPGA compilation tools, where some even have interfaces from higher level languages like *C* or *Simulink*. Such tools even exist for analog signal processing compilation, such as the tool suite controlled through MATLAB (Koziol et al., 2010), using Simulink (Schlottmann et al., 2012a) at the high level that compiles to a spice deck, which in turn, can be compiled (Baskaya et al., 2009) to programmable object code for the FPAA device. Higher-level tools also enable the use of these systems in educational experiences (Twigg and Hasler, 2008), which will be essential to educating engineers to *design* with neuromorphic concepts for system applications that are superior to state of the art solutions.

In the literature, we find a large number of proposed tools, typically being used by a few computational neuroscientists, each being rewritten for a particular feature, or concept. Examples of these tools would include PyNN (Davison et al., 2008), written in Python, and JAER (jAER, 2011), based on Java and connects to Python interfaces. Further, there are classic neural computation tools such as Genesis (Bower and Beeman, 1998), neuron (Hines and Carnevale, 1997), and bib48 (Goodman and Brette, 2008) which have wide applicability, and are known to be useful at different levels of abstraction/computation for the resulting ODE solutions required.

Unfortunately, there are few approaches that attempt to bridge across a range of approaches, in particular, tools used by multiple computational groups as well as multiple hardware groups. The notable exception is the PyNN tool, originating from the Heidelberg group (EU FACETs program) which shows promise for a tool to unify multiple groups through an open community type tool used by multiple academics. PyNN is designed to be a simulator-independent, Python-based open source language designed for describing spiking neural network models. PyNN is the one tool that is currently used for multiple heterogeneous neuron platforms. For example, the FPAA tool flow shows initial tools (Schlottmann et al., 2012a) that could also utilize a PyNN structure to compile to hardware. The base language we used for this approach is PyNN (Davison et al., 2008), rather than a spice deck, to specify the netlist level of the neuron structure. Extending PyNN as a tool for design approaches would move further along this goal.

## Overview of adaptation and learning

In this section, we give an overview considering adaptation and learning in this hardware roadmap. Because learning function, not to mention computation, is an open area of research, the ability to predict potential long-term issues is challenging. We have some visability into the device-level issues for adaptation and learning, programming versus learning for an entire array, as well as some development questions for learning synaptic elements; we will consider each of these in the following subsections. We see key issues for learning and adaptation to address going forward

- FG approaches sets the standard for a single 3-terminal device providing integrated (non-volatile) memory, synapse density, resolution (digital EEPROM store 4 bits/cell at 22 nm), low-power, and local adaptation. Easy local control and mismatch control are nice to have features.
- Development/Investigation of system level (groups of event neurons) learning rules, including normalization of neuron/synaptic activity.
- Neuron learning utilizing dendritic structure. Recent results on dendritic computation gives hope to understand algorithmic issues. Circuit approach requires dense circuit models in configurable architectures.
- Axon routing as well as slower timescale chemical changes could further add capability, particularly once key neuron learning aspects are stable.

### Device-level questions for adaptation and learning

Device-level neural system learning starts looking at synapse circuit models, as well as finding approaches to implement these functions using as little additional synapse circuitry as possible to enable tight computation. One metric of a learning model is quantifying (and minimizing) the percentage increase in base synapse cell size from an programmable synapse to an adaptive synapse. The floating-gate based learning structures, single transistor learning synapses (STLS) (Hasler et al., 1995), the floating-gate LMS adaptive filter (Hasler and Dugger, 2005), and floating-gate STDP synapses (Ramakrishnan et al., 2011; Brink et al., 2012; Nease et al., 2013), all show this overhead metric is manageable and approaches zero in some cases; the cell size is relative to EEPROM type devices, with the size, complexity, IC processing, and manufacturing benefits mentioned earlier. The LMS structure increases the cell size over a VMM structure (Schlottmann and Hasler, 2011) by a factor of roughly 2, and the STDP synapse structure size (Ramakrishnan et al., 2011) is identical to the resulting floating-gate transistor-channel model (Gordon et al., 2004). Mesh-type configurations are good for synaptic arrays when the dendrites are considered wires even when utilizing learning in the network, with additional circuit control on the periphery of the array. Further, other parameters such as additional power dissipation and added noise should be low relative to the non-adapting computation, often seen in floating-gate based approaches (Hasler et al., 1996; Hasler and Dugger, 2005).

Some nanotechnology elements, such as memristors, also have a clear multiple-timescale behavior that would enable potentially adaptation and long-term storage in a single device. Widrow's original adaptive filter work was performed by what he called three-terminal memristors (Widrow, 1960); enabling learning function in two terminal memristors is a challenge because in a mesh (crossbar) array it is hard to get desired functionality, although some early simulation results showing the approach might the possible (Zamarreo-Ramos et al., 2011). What is also likely with similar nano device structures is to enable circuit elements that can modulate a conductance on a slow-timescale based on network dynamics, in a dense structure, potentially integrated above the Si IC. Neuroscience uses a wide range of timescales for its computation and learning requiring we eventually need these mechanisms (Sejnowski and Churchland, 1992).

Introduction of dendritic structure, motivated by previous sections for its computational importance and efficiency, significantly changes the elegant mesh array of synaptic devices. Dendrites add complexity both in terms of required added circuitry as well as potentially additions to the learning algorithms, such as requiring local Ca^2+^ and localized synaptic learning, where the detailed biological modeling in these areas are still open questions. A dense configurable array of adapting synapses with dendritic reconfigurability still enables these approaches, even with the ever improving research in this area.

### Writing/reading synapse values from a cortical model

If the synapse strengths/weights are learned, this alleviates the need for loading a large number of parameter values into a system. Assuming we are loading a cortex of 10^15^ synapses, this requires significant communication time and overall system power. Table 4 shows the cost and complexity for communicating the resulting digital values to the synapses. The computations use 10 bit accuracy for the device values, 300 pF system load capacitance, and V_*dd*_ at 2.5 V. We expect to have many parallel input data streams to load the entire array for a sustained rate of 11.3 Tbit/s, probably coming from multiple memory sources to hold the 1000 TByte golden memory target. These issues are typical for loading a supercomputer system (TOP500 List, 2012). We have a similar issue for reading the network; reading the entire state of the weights (and/or all potentials) once is an expensive proposition.

**Table 4:** **Summary table for loading synapses in a human brain (10^15^)**.

**Load time**	**15 min**	**1 day**	**10 days**
Communication			
Rate	11.3 Tbit/s	116 Gbit/s	11 Gbit/s
Power	10.4 kW	109 W	11 W

Loading a single IC with 10^9^ synapses (say 10^6^ neurons) in a second would require 10 Gbit/s data link into the IC requiring 1.6 W for communication for a 50 pF load (minimum level for IC test with zero-insertion force socket). The challenge of parallel programming these number of synapses on chip is managable, and the resulting power requirements are significantly less than the data communication. These numbers directly impact the final cost of such a system; IC testing can be a significant cost in manufacturing of a final product; loading values in 1 s prevents one such product limitation. For the 10^15^ synapse data loading the power consumption and performance will be limited by the system communication, not the IC complexity.

For a 20 W system, loading the weights frequently is not possible; this point further illustrates the untenable case of storing synapse weights in one place and using them somewhere else, even in a multiplexed system. Once a memory is programmed, adapted, and/or learned, reloading the memory is costly; therefore, non-volatile memory is critical to minimize the cost of loading a system. On the other hand, occasionally loading an entire cortex of 10^15^ synapses, say on the order of once a day, is a feasible proposition, as well as having programmed code at the initial condition or reset condition for a commercial machine.

One might wonder if every synaptic weight, as well as every neuron parameter, can be learned or adapted from the resulting environment. History developing with adaptive systems, both non-spiking (Hasler and Dugger, 2005) and spiking (Brink et al., 2012; Nease et al., 2013), required some precisely programmed elements, although fewer than the total number of learned parameters. Often these programmed parameters should be insensitive to environmental conditions, often requiring a few precision current and voltage sources. The programming of these few parameters often have a large effect on the resulting algorithm behavior. This behavior leads one to speculate whether the brain uses the precise data from the human genome, estimated to be roughly 3.2 billion base pairs long contain 20,000–25,000 distinct genes represented by 800 Mbytes of data (International Human Genome Sequencing Consortium, 2004; Christley et al., 2008) to set the behavior in a similar way the parameters of billions of neurons and 10^15^ synapses.

### Thoughts on learning and development of neuron arrays

One classic question for biological learning networks is how the synapses from an array of neurons, say from one or multiple layers in cortex, would converge to a system equilibrium to investigate the resulting functions of the neuron array, and compare with biological studies. Several fundamental studies exist in this area treating neurons as an ANN type model with different learning rules finding patterns corresponding to Principle Component Analysis (PCA) (e.g., Linsker, 1988; MacKay and Miller, 1990), Independent Component Analysis (ICA) (e.g., Bell and Sejnowski, 1995, 1997; Hoyer and Hyvarinen, 2000), and a range of modified approaches based on this work (Blais et al., 1998; Zylberberg et al., 2011; Falconbridge et al., 2006; Saxe et al., 2011). These approaches are built around fundamental continuous-time ANN algorithms on PCA algorithms (Oja, 1982; Sanger, 1989) as well as ICA built from non-linearities (Hyvärinen and Oja, 1997), each with grounding to talk about potential computation and applications coupled with approaches to build such algorithms (Cohen and Andreou, 1992, 1995; Hasler and Akers, 1992).

The fundamental issue is the difficulty of making such progress with spiking neurons. The lack of computational models in spiking networks, including representations of events and resulting realistic sensory data, complicates the analysis of the resulting learning network. Most learning experiments use encoding structures that reduce the network (e.g., Savin et al., 2010), although they recognize issues of rate encoding, reducing many of the results to ANN approaches.

The case becomes even less studied when considering realistic dendritic structures. Development with dendrites with spike representation is an open question, and an exciting area of research. Early research on the wordspotting dendritic computation with STDP learning has some similarity to HMM learning rules, but the careful connection is yet to be understood. Further questions come from understanding and implementing the development axon growth/routing algorithms used in development, particularly as implemented in hardware (Boerlin et al., 2009). Some evidence exists that dendritic activity strongly affects the directions of the axonal projections (e.g., Richardson et al., 2009). We expect wide-open opportunities as well as high-impact results coming from investigations in this area.

## Conclusions

**Figure 25:**
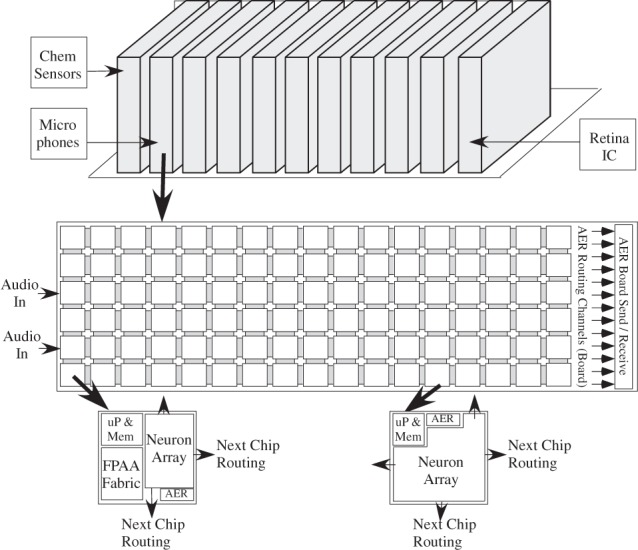
**A potential view of how one could build a brain/cortical structure; the approaches follow constraints outlined throughout this discussion.** The approach could be integrated as a set of boards with a large number of neural ICs, where at each level of complexity, local communication is emphasized for power efficient computation as well as low integration complexity. Most of the on-chip communication would be local, most of the chip-to-chip communication would be between neighboring ICs in an extended FPGA like fabric. The system would Interface to typical biological sensors, like retina (vision), microphones for audition, and chemical sensors, as well as non-biological (i.e., communication spectrum) inputs. A particular neuron array could be integrated with additional FPAA structures enabling integration of analog SP for the front-end processing (i.e., acoustic front-end processing).

This study concludes that useful neural computation machines based on biological principles at the size of the human brain seems technically within our grasp. Building a supercomputer like structure to perform computations in human cortex is within our technical capability, although more a question of funding (research and development) and manpower. Figure 25 shows a representative cortical system architecture of silicon neuron structures. The heavy emphasis on local interconnectivity dramatically reduces the communication complexity. We show these capabilities are possible in purely CMOS approaches, not necessarily relying on novel nanotechnology devices.

**Figure 26:**
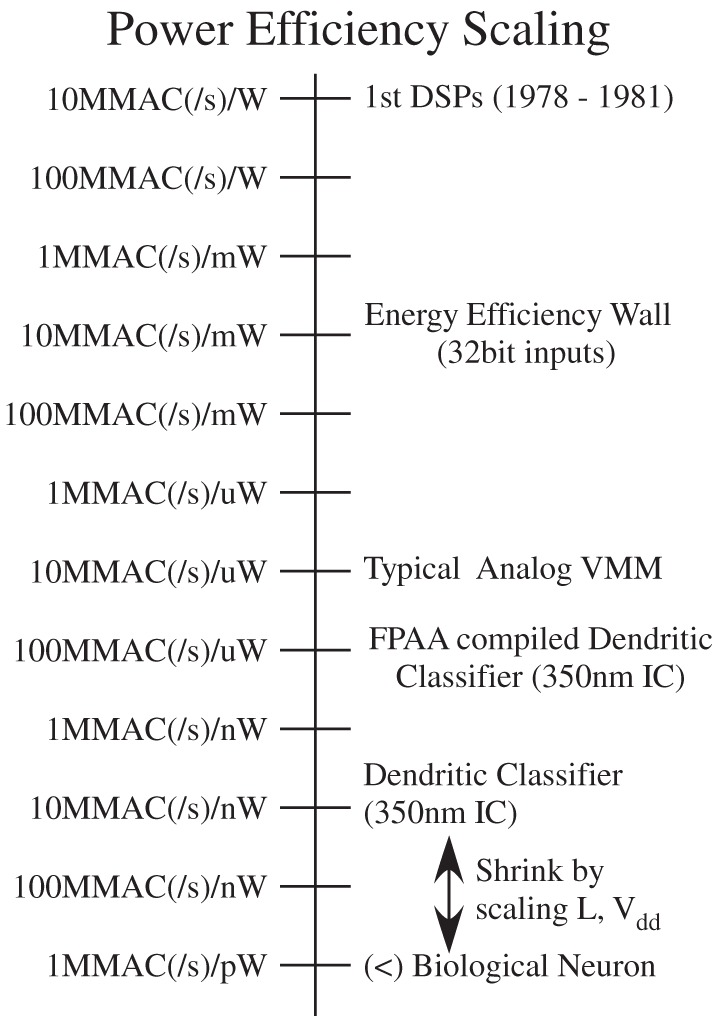
**A summary comparison of power efficient computational techniques, including digital, analog Signal Processing (SP) techniques, and the potential for neuromorphic physical algorithms.** The potential of 8–9 orders of magnitude of achievable computational efficiency encourages a wide range of neuromorphic research going forward.

Figure 26 shows the potential computational energy efficiency in terms of computation for digital systems, analog signal processing, and potential neuromorphic hardware-based algorithms. Computational power efficiency for biological systems is 8–9 orders of magnitude lower than the power efficiency wall for digital computation; analog techniques at a 10 nm node can potentially reach the same level of computational efficiency. The resulting tradeoffs show that a purely digital circuit approach are less likely because of the differences in computational efficiency. These approaches show huge potential for neuromorphic systems, showing we have a lot of room left for improvement (Feynman, 1960), as well as potential directions on how to achieve these approaches with technology already being developed; new technologies only improve the probability of this potential being reached.

**Figure 27:**
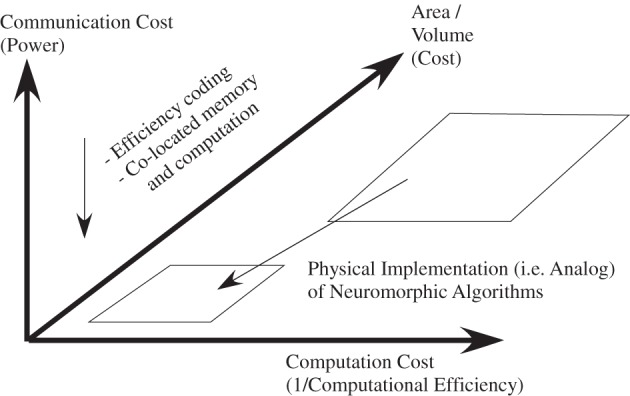
**Overview figure illustrating the three dimensions (computational efficiency, communication power, and system area) to optimize to reach large-scale neuromorphic systems.** Using physical based (i.e., analog) approaches help to decrease computational efficiency and system area, and heavy use of local communication, integration of memory and computation, as well as low-event architecture reduces the communication power required.

Figure 27 illustrates the key metrics of computational efficiency, communication power, and system area. Physical computing, based on neuromorphic concepts, potentially can dramatically improve system area and computational efficiency, as illustrated throughout this discussion. Understanding that the nervous system is power constrained is not only a key technological parameter, but understanding its implication for communication enables building systems that won't be handicapped by its control infrastructure. This comparison requires keeping communication local and low event rate, two properties seen in cortical structures. Communication power efficiency is handled by minimizing long-distance communication events, focusing architectures on local communication, and refining data to minimize the number of long-distance events communicated. These points give some metrics for successful neuromorphic systems, in particular how much improvement in power efficiency achieved compared to a standard analog signal processing approach.

Probably the largest hurdle is not about what we can build, but identifying novel, efficient computation in neurobiology and employing these techniques in engineering applications. This question is the fundamental open question for neuromorphic engineering as well as neuroscience. Given that the neuromorphic engineering building blocks also can be accurate models for neurobiological behavior, these questions are directly related. We painted a picture of the potential computational models arising from neuro-modeling, including their potential computational efficiency; we expect these models are just a start to what is possible. We expect neuroscientists are bound to make more fundamental discoveries about the nature of the biological computation, discoveries that most likely will further improve the computational efficiency and other metrics of these systems.

Finally, the research in this area will accelerate by the pull of commercial ventures that can start utilizing these technologies to competitive commercial advantage. The pull of commercial success, particularly if ICs are available, will rapidly help advance the pace of neuromorphic engineering and computational neuroscience.

### Conflict of interest statement

The authors declare that the research was conducted in the absence of any commercial or financial relationships that could be construed as a potential conflict of interest.
